# Skin organoid transplantation promotes tissue repair with scarless in frostbite

**DOI:** 10.1093/procel/pwae055

**Published:** 2024-10-04

**Authors:** Wenwen Wang, Pu Liu, Wendi Zhu, Tianwei Li, Ying Wang, Yujie Wang, Jun Li, Jie Ma, Ling Leng

**Affiliations:** Stem Cell and Regenerative Medicine Lab, Institute of Clinical Medicine, State Key Laboratory of Complex Severe and Rare Diseases, Peking Union Medical College Hospital, Chinese Academy of Medical Sciences and Peking Union Medical College, Beijing 100730, China; State Key Laboratory of Medical Proteomics, Beijing Proteome Research Center, National Center for Protein Sciences (Beijing), Beijing Institute of Lifeomics, Beijing 102206, China; Chongqing Key Laboratory on Big Data for Bio Intelligence, Chongqing University of Posts and Telecommunications, Chongqing 400065, China; Stem Cell and Regenerative Medicine Lab, Institute of Clinical Medicine, State Key Laboratory of Complex Severe and Rare Diseases, Peking Union Medical College Hospital, Chinese Academy of Medical Sciences and Peking Union Medical College, Beijing 100730, China; State Key Laboratory of Medical Proteomics, Beijing Proteome Research Center, National Center for Protein Sciences (Beijing), Beijing Institute of Lifeomics, Beijing 102206, China; Stem Cell and Regenerative Medicine Lab, Institute of Clinical Medicine, State Key Laboratory of Complex Severe and Rare Diseases, Peking Union Medical College Hospital, Chinese Academy of Medical Sciences and Peking Union Medical College, Beijing 100730, China; Stem Cell and Regenerative Medicine Lab, Institute of Clinical Medicine, State Key Laboratory of Complex Severe and Rare Diseases, Peking Union Medical College Hospital, Chinese Academy of Medical Sciences and Peking Union Medical College, Beijing 100730, China; Stem Cell and Regenerative Medicine Lab, Institute of Clinical Medicine, State Key Laboratory of Complex Severe and Rare Diseases, Peking Union Medical College Hospital, Chinese Academy of Medical Sciences and Peking Union Medical College, Beijing 100730, China; State Key Laboratory of Medical Proteomics, Beijing Proteome Research Center, National Center for Protein Sciences (Beijing), Beijing Institute of Lifeomics, Beijing 102206, China; Stem Cell and Regenerative Medicine Lab, Institute of Clinical Medicine, State Key Laboratory of Complex Severe and Rare Diseases, Peking Union Medical College Hospital, Chinese Academy of Medical Sciences and Peking Union Medical College, Beijing 100730, China

**Keywords:** skin frostbite, frostbite treatment, human-induced pluripotent stem cell, skin organoids, single-cell transcriptomics

## Abstract

Frostbite is the most common cold injury and is caused by both immediate cold-induced cell death and the gradual development of localized inflammation and tissue ischemia. Delayed healing of frostbite often leads to scar formation, which not only causes psychological distress but also tends to result in the development of secondary malignant tumors. Therefore, a rapid healing method for frostbite wounds is urgently needed. Herein, we used a mouse skin model of frostbite injury to evaluate the recovery process after frostbite. Moreover, single-cell transcriptomics was used to determine the patterns of changes in monocytes, macrophages, epidermal cells, and fibroblasts during frostbite. Most importantly, human-induced pluripotent stem cell (hiPSC)-derived skin organoids combined with gelatin-hydrogel were constructed for the treatment of frostbite. The results showed that skin organoid treatment significantly accelerated wound healing by reducing early inflammation after frostbite and increasing the proportions of epidermal stem cells. Moreover, in the later stage of wound healing, skin organoids reduced the overall proportions of fibroblasts, significantly reduced fibroblast-to-myofibroblast transition by regulating the integrin α5β1-FAK pathway, and remodeled the extracellular matrix (ECM) through degradation and reassembly mechanisms, facilitating the restoration of physiological ECM and reducing the abundance of ECM associated with abnormal scar formation. These results highlight the potential application of organoids for promoting the reversal of frostbite-related injury and the recovery of skin functions. This study provides a new therapeutic alternative for patients suffering from disfigurement and skin dysfunction caused by frostbite.

## Introduction

Throughout history, frostbite has threatened people who live or explore plateaus in alpine regions (Castellani and Young, 2012; Fudge et al., 2015; Harirchi et al., 2005; Heil et al., 2016), and preventing frostbite has always been a significant focus of the military (Sokolov et al., 2017). Moreover, frostbite can also occur in urban areas, where poor social status, physical disability, homelessness, and mental illness represent risks for freezing injury (Hallam et al., 2010; Lindford and Vuola, 2011; Sheridan et al., 2022). Importantly, the initial stage of low-temperature injury is insidious, and symptoms are easily ignored by patients and clinicians (Johnson-Arbor, 2014; Sokolov et al., 2017; Tobalem et al., 2010), which often leads to serious consequences. The skin is the largest organ of the human body and the first barrier against external mechanical or chemical damage and invasion by pathogens (Gravitz, 2018; Li et al., 2022). Frostbite occurs when the skin is directly exposed to low temperatures and is most common in exposed areas of the body, including the hands, feet, cheeks, ears, and nose (Harirchi et al., 2005). Patients with severe skin frostbite require amputation (Dempsey et al., 2018; Johnson-Arbor, 2014), and many patients with frostbite often suffer from nonunion, scarring, chronic pain, and dysfunction (Graham and Stevenson, 2000; Sheridan et al., 2009, 2022).

Generally, skin frostbite can cause direct and indirect damages. Direct damage is the direct effect of cold on the tissue, leading to ice crystal formation, temperature-induced protein changes, and membrane damage, which result in the destruction of hair follicles and sebaceous glands and necrosis of epidermal and dermal cells (Biem et al., 2003; Lindford and Vuola, 2011). Indirect injury results from insufficient blood supply caused by vasoconstriction, endothelial damage, and inflammatory mediators such as thromboembolism, prostaglandins, and oxygen radicals (Sheridan et al., 2009). Therefore, injuries caused by frostbite are more complex and more difficult to heal than ordinary skin injuries. There is currently no effective treatment for frostbite (Biem et al., 2003; Sheridan et al., 2009). Calcium channel blockers (especially nifedipine) appear to be the most effective current treatment for frostbite. Their mechanism of action involves dilating capillaries at the frostbite site and improving microcirculation, which indirectly affects skin cells and the extracellular matrix (ECM). They are best used immediately after frostbite occurs, preferably as early as possible, as their efficacy is diminished when applied late in frostbite progression after vascular paralysis and necrosis have occurred. Furthermore, they act indirectly to improve the ischemic-hypoxic state of frostbitten skin cells rather than directly on them, resulting in a slow onset of action and delayed wound closure. Such delayed healing wounds are prone to infection and hinder collagen remodeling, leading to long-term scar formation. Additionally, necrotic cells, especially stem cells in the skin, cannot regenerate through this indirect improvement of microcirculation. Over the past two decades, there have been extensive developments in the field of wound healing (Cao et al., 2023; Tatara et al., 2018; Wang et al., 2022), Stem cell therapy has attracted considerable attention for its multiple advantages, particularly in promoting rapid wound healing and epidermal regeneration (Chen et al., 2024; Shang et al., 2024; Sun et al., 2024; Wang et al., 2024). Stem cells can stimulate the growth and migration of epithelial cells, facilitate the reconstruction of the epidermal barrier, and accelerate the process of re-epithelialization in damaged tissues (Li et al., 2024; Shang et al., 2024). However, the cell types used in current stem cell therapies are relatively limited, mainly including mesenchymal stem cells, hematopoietic stem cells, neural stem cells, etc. (Ferrari et al., 2023; Hosseini et al., 2024; Jo et al., 2021). Additionally, the challenge of non-scarring wound healing after trauma remains unresolved by stem cell therapy.

Tissue repair is a highly complex event involving a series of continuous coordinated signals and responses from fibroblasts, epithelial cells, endothelial cells, and immune cells (Nie et al., 2024). Studies have shown that these different molecular events all involve the presence of the ECM (Chavez and Gerecht, 2023; Hao et al., 2022). The precise remodeling process involved in skin wound healing requires a good balance between ECM deposition and degradation, particularly in the dermal compartment, where fibroblasts and myofibroblasts play a central role. Myofibroblasts can regulate wound contraction by secreting ECM, increasing wound tension, and affecting the degree of fibrosis in the wound, with excessive fibrosis often leading to the formation of hypertrophic scars (Ackerman et al., 2024; Schuster et al., 2023). Furthermore, disruption or delay in epithelialization during the wound-healing process can increase the frequency of chronic wounds or fibrotic conditions, leading to the formation of pathological scars in the later stages (Amiri et al., 2022). Although regeneration is the holy grail of tissue repair, skin injuries often result in fibrotic, nonfunctional scars. Unfortunately, the problem of scarless regeneration has not yet been solved (Zhou et al., 2023).

With the development of the field of stem cells, research on organoids is also advancing continuously. Various organoids consist of multiple cell types and possess similar spatial organization and partial functions of corresponding organs (Maharjan et al., 2024). In the application field of skin organoids, researchers have used skin organoids to investigate the pathogenesis of monkeypox virus, coronavirus in the skin, and evaluate antiviral drugs (Li et al., 2023a; Ma et al., 2022b). This indicates that the skin organoid model system has great potential in exploring virus–host interactions and testing antiviral drugs. However, the potential role of skin organoids with multiple cell types in wound repair is not yet clear. Here, we generated human-induced pluripotent stem cell (hiPSC)-derived skin organoids with skin appendages and complete neuronal circuits according to the previous method (Ma et al., 2022a, 2022b) and used them to treat full-thickness wounds resulting from frostbite in nude mice to provide evidence for the use of skin organoids to treat irreversible skin damage.

## Results

### Pathological and single-cell transcriptome analyses of skin affected by frostbite in mice

**Figure 1.**
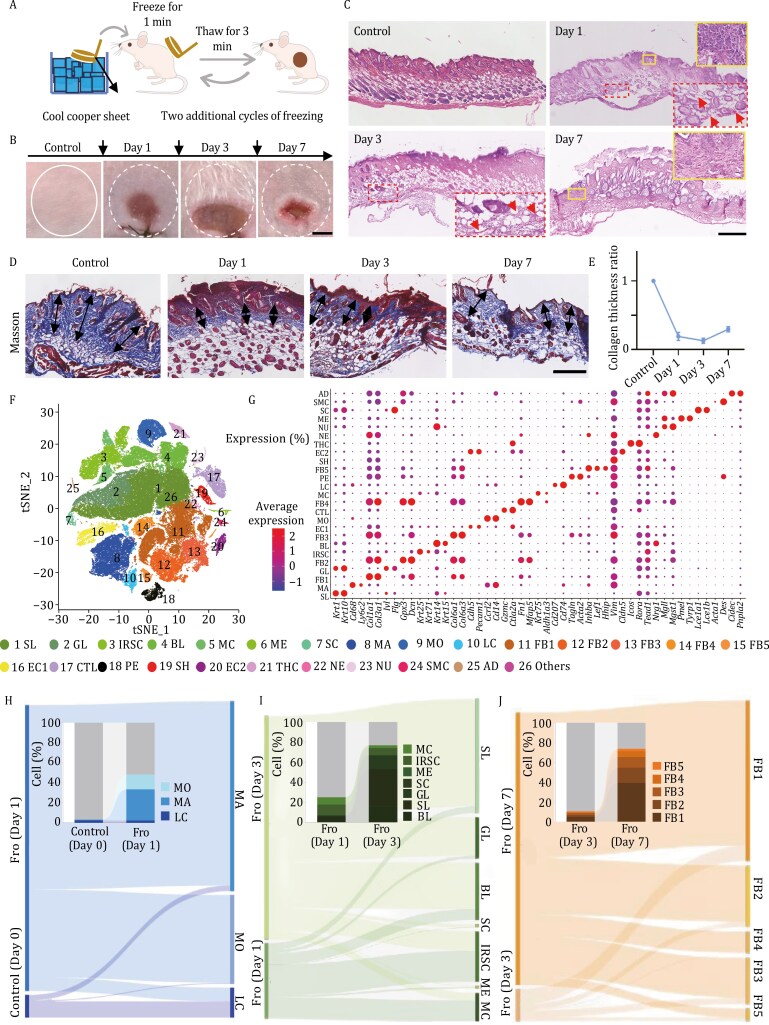
Pathological and single-cell transcriptomic analysis of the skin of frostbite model mice. (A) Workflow of frostbite injury modeling in mice. A copper sheet was frozen to approximately −80°C using dry ice and applied to the dorsal skin of mice for 1 min. Subsequently, the copper sheet was removed, allowing the frozen skin to thaw for 3 min. This procedure was repeated three times in total. (B) Images of normal skin of control mice and frostbite-related wounds on days 1, 3, and 7 (scale bar: 2 mm). (C) Hematoxylin and eosin (H&E) staining of the normal skin of control mice and frostbite model mice at days 1, 3, and 7 (scale bar: 500 µm). The solid lines on day 1 images indicate inflammatory cells, the dashed lines on day 1 and 3 images indicate capillary ectasia and erythrocyte stasis with the arrows indicateing disrupted blood vessels, and the dashed lines on day 7 images indicate an increased number of fibroblasts in the dermis. The experiment was repeated three times. (D) Masson staining of normal skin of control mice and frostbite-affected skin on days 1, 3, and 7 (scale bar: 200 µm). The black arrow shows the collagen thickness. The experiment was repeated three times. (E) Analysis of the collagen thickness ratio in the skin of frostbite model mice on days 0, 1, 3, and 7. (F) tSNE (t-distributed stochastic neighbor embedding) plots of scRNA-seq data generated from the skin samples of control and frostbite model mice. The major cell groups were manually annotated and are labeled with different colors. (G) Expression of representative marker genes for each cell type. The statistical significance of cell type marker genes was determined using the Wilcoxon test with a Benjamini–Hochberg (BH)-adjusted *P* value < 0.01. The proportions of immune cells (H), epidermal cells (I), and fibroblasts (J) in normal control and frostbite model mice on different days. The differently colored blocks of histograms represent the proportions of different cell types. Fro: frostbite; SL: spinous layer cell; GL: granular layer cell; IRSC: inner root sheath cell; BL: basal layer cell; MC: medulla cell; ME: melanocyte; SC: stratum corneum cell; MA: macrophage; MO: monocyte; LC: Langerhans cell; FB1: fibroblast 1; FB2: fibroblast 2; FB3: fibroblast 3; FB4: fibroblast 4; FB5: fibroblast 5; EC1: endothelial cell 1; CTL: cytotoxic T lymphocyte; PE: pericyte; SH: Schwann cell; EC2: endothelial cell 2; THC: T helper cell; NE: neuron; NU: neutrophil; SMC: skeletal muscle cell; AD: adipocyte.

To investigate the mechanism of frostbite healing, we constructed a mouse model of frostbite-induce full-thickness wounds using a freeze‒thaw‒freeze cycle (Fig. 1A). We found that the skin of frostbite model mice was darker red than that of normal mice (Fig. 1B). At 24 h after frostbite, the skin was swollen and bruised (Fig. 1B), and pathological analysis revealed many inflammatory cells (monocytes) in both the dermis and epidermis (Fig. 1C). At 3 days after injury, the wounds were the largest and most severe (Fig. 1B and 1C), which is consistent with previous reports (Auerbach et al., 2013). Moreover, 3 days after frostbite, the capillaries were dilated, and many red blood cells accumulated in the vascular lumens (Fig. 1C). Until day 7, accumulation of many fibroblasts was observed. These cells were potentially preparing for remodeling of the ECMs (Fig. 1C). Masson staining revealed that the expression of collagen decreased sharply at 1 day after frostbite and then slowly recovered by 7 days (Fig. 1D and 1E), indicating that frostbite severely affected the expression and distribution of ECM components.

To further investigate the pattern of cellular changes in skin tissues after frostbite, single-cell transcriptomics analysis was performed to analyze the proportions of different skin cell types in mice at different time points after frostbite. The results revealed 25 cell types in mouse tissue, including epithelial cells (49.06%, including basal layer cells, spinous layer cells, granular layer cells, stratum corneum cells, melanocytes, inner root sheath cells, and medulla cells), immune cells (15.51%, including Langerhans cells, macrophages, and monocytes), 5 types of fibroblasts (24.45%, fibroblasts 1–5), and other cells (Figs. 1F, 1G, S1A, S2, S3, and Table S1). We found that the proportions of immune cells, including monocytes and macrophages, increased significantly at 1 day (48.2%) after frostbite compared to normal mouse skin tissues (3.3%), while the proportion of Langerhans cell (control: 2.4%, frostbite day 1: 2.3%) did not change much (Fig. 1H). These findings indicate that monocytes and macrophages play important roles in promoting the immune response in the initial stage of tissue repair after frostbite. Furthermore, we analyzed the three subtypes of monocytes including classical monocytes (*Cd14++*), intermediate monocytes (*Cd14+*), and non-classical monocytes (*Cd14*−), and the two subtypes of macrophages including M1 macrophages (*Cd68+*) and M2 macrophages (*Cd206+*) (Fig. S1B–D). We observed a substantial increase and decrease in the proportion of intermediate and classical monocytes (from 6.1% to 97.2%) and non-classical monocytes (from 93.9% to 2.8%) in mouse skin at 1 day after frostbite compared to the normal controls, respectively (Fig. S1E). Additionally, the proportion of M2 macrophages increased slightly in mouse skin at 1 day after frostbite (from 81.1% to 86.6%) (Fig. S1F). These results indicate that the non-classical monocytes may predominantly transfer to intermediate and classical monocytes, and M2 macrophages could be the dominant type of the increased macrophages in mouse skin at 1 day after frostbite compared to the normal samples.

We also found that the proportions of epithelial cells including basal layer cells, spinous layer cells, granular layer cells, stratum corneum cells, and melanocytes increased greatly on day 3 compared to day 1 after frostbite; however, the proportions of inner root sheath cells and medulla cells did not increase (Fig. 1I), indicating that the proportions of epithelial cells may increase rapidly at day 3 after frostbite due to the need for re-epidermalization. In addition, we found that the proportions of five types of fibroblasts increased at day 7 compared to day 3 after frostbite (Fig. 1J), indicating that fibroblasts may secrete ECM components for remodeling of the ECM microenvironment at day 7 after frostbite.

In summary, we successfully constructed a frostbite-induced skin injury model that exhibited dynamic changes in chronological order. Single-cell transcriptome profiling of the frostbite model was performed to determine the differences in the proportions of skin cell types and their distribution following frostbite. Next, the single-cell transcriptome atlas was used to study the functional changes in different skin cell types after frostbite.

### Analysis of gene expression changes in different cell types at the single-cell level following frostbite

**Figure 2.**
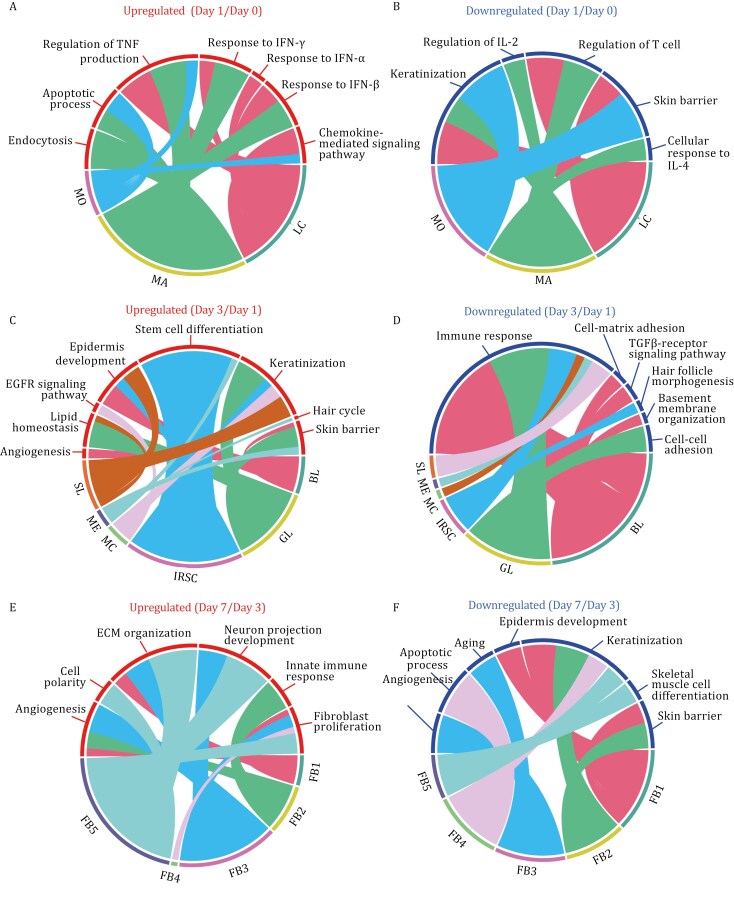
Functional analysis of upregulated and downregulated genes in different types of cells between the control and frostbite groups. Functional analysis of upregulated and downregulated differentially expressed genes (DEGs) in immune cells (A and B), epidermal cells (C and D), and fibroblasts (E and F) in mouse skin samples from the control and frostbite groups on different days. The immune cell subtypes included Langerhans cells, monocytes, and macrophages; the epidermal cell subtypes included basal layer cells, spinous layer cells, granular layer cells, stratum corneum cells, melanocytes, inner root sheath cells, and medulla cells; and there were five fibroblast subtypes: fibroblast 1, fibroblast 2, fibroblast 3, fibroblast 4, and fibroblast 5. Genes with a Benjamini-Hochberg (BH)-adjusted *P* value < 0.05 and log_2_FC > 0.25 (upregulated) or < −0.25 (downregulated) were identified as DEGs. The color of the upper half of the ring indicates whether the biological processes were enriched for upregulated or downregulated DEGs, respectively; the color of the lower half of the ring indicates the different cell type. SL: spinous layer cell; GL: granular layer cell; IRSC: inner root sheath cell; BL: basal layer cell; MC: medulla cell; ME: melanocyte; SC: stratum corneum cell; MA: macrophage; MO: monocyte; LC: Langerhans cell; FB1: fibroblast 1; FB2: fibroblast 2; FB3: fibroblast 3; FB4: fibroblast 4; FB5: fibroblast 5.

To investigate the changes in gene expression in different cell types after frostbite, the differentially expressed genes (DEGs) were identified in immune cells at day 1 compared with day 0 after frostbite, epithelial cells at day 3 compared with the day 1 after frostbite, and fibroblasts at day 7 compared with the day 3 after frostbite. The results revealed 1,296, 749, and 1,626 upregulated genes and 493, 779, and 371 downregulated genes for immune cells, epithelial cells, and fibroblasts at day 1, 3, and 7 after frostbite, respectively (Tables S2–5). The upregulated genes in macrophages at day 1 after frostbite were enriched mainly in the biological processes of endocytosis (*App*, *Rab1a*, etc.), regulation of TNF production (*Fcr1g*, *Syk*, etc.), and the responses to IFNγ (*Stat1*, *Ccl9*, etc.) and IFNβ (*Ifnar2, Ifi209,* etc.). The genes upregulated in monocytes were enriched mainly in chemokine signaling (*Ccr1*, *Ccrl2*, etc.) and TNF production regulation (*Fcer1g*, *Syk*, etc.) (Fig. 2A). On the other hand, the genes downregulated in macrophages were enriched mainly in the regulation of interleukin 2 (IL2) production (*Cd83*, *Irf4*, etc.) and T cells (*H2dmb2*, *H2eb1*, etc.), as well as the response to interleukin 4 (IL4) (*Dcstamp*, *Rpl3*, etc.) (Fig. 2B). Although the proportion of Langerhans cells changed only slightly at day 1 after frostbite, the downregulated genes in these cells were enriched mainly in the skin barrier (*Flg*, *Sfn*, etc.) and keratinization (*Sprr2f*, *Sprr2h*, etc.) (Fig. 2B). These results suggested that monocytes and macrophages are mainly responsible for the regulation of inflammatory factor release and the inflammatory factor response at 1 day after frostbite. Further, we analyzed the DEGs of monocytes and macrophages at days 3 and 7 after frostbite (Fig. S4). The DEGs of monocytes at day 3 after frostbite were still enriched in the inflammation-related functions (Fig. S4A), and more DEGs were enriched in the functions of cell migration and tissue remodeling in the later stage of frostbite (Fig. S4B). Meanwhile, the functions of DEGs of macrophages changed from strong inflammatory response and immune cell recruitment to the processes of antigen processing and presentation, immune regulation, and tissue repair at days 3 and 7 after frostbite (Fig. S4C and S4D).

Next, we analyzed the biological functions of DEGs in different types of epithelial cells at day 3 compared with day 1 after frostbite. As expected, upregulated genes in cells in the basal layer, i.e., stem/progenitor cells and melanocytes were enriched in epidermal development (*Krtdap*, *Krt79*, etc.), stem cell differentiation (*Krt10*, *Epcma*, etc.) and the skin barrier (*Krt1*, *Cldn1*, etc.); upregulated genes in cells in the spinous layer were enriched in epidermal development (*Ktr10*, *Cdkn1a*, etc.) and keratinization (*Krt1*, *Krt16*, etc.); and upregulated genes in cells in the granular layer were enriched in keratinization (*Krt80, Krt77*, etc.), the skin barrier (*Flg*, *Elov1l*, etc.) and lipid homeostasis (*Alox12b*, *Abhd5*, etc.) (Fig. 2C). These results illustrate the contribution of different epidermal cell layers to re-epidermalization within 3 days after frostbite. Interestingly, the genes upregulated in inner root sheath cells were enriched mainly in stem cell differentiation, suggesting that activated inner root sheath cells might contribute to re-epidermalization at 3 days after frostbite (Fig. 2C). On the other hand, we found that the genes downregulated in basal layer and granular layer cells were enriched mainly in the immune response (*Il1b*, *Il1rl1*, etc.), indicating that keratinocytes exhibit only mild involvement in inflammatory responses at 3 days after frostbite compared to 1 day. In addition, the expression of basement membrane-associated genes (*Col4a1*, *Itgb1*, etc.) in the basal layer, as well as cell adhesion genes (*Dsp*, *Dsg3*, etc.) in the granular layer, decreased at 3 days after frostbite 3 days (Fig. 2D), indicating dynamic changes in the basement membrane, cell–matrix junction components, and keratinocytes during re-epidermalization.

Then the functions of five fibroblast-associated DEGs at day 7 compared to day 3 after frostbite were analyzed. The genes upregulated in fibroblasts 1, 3, and 5 were enriched mainly in matrix assembly (*Col1a1*, *Col3a1*, etc.) and neurodevelopment (*Sox11*, *Plxna4*, etc.), indicating that these fibroblasts are responsible for ECM component expression and ECM remodeling, as well as the regulation of never system (Fig. 2E). The genes upregulated in fibroblasts 2 were enriched in innate immune responses (*Ifi204*, *Ifi202b*, etc.), and the genes upregulated in fibroblasts 4 and 5 were enriched mainly in fibroblast proliferation (*Wapl*, *Ddr2*, etc.) (Fig. 2E). These findings indicate that there is a subset of fibroblasts that may respond to immune signals and that a portion of cells in this subset need to maintain self-renewal capacity. The genes downregulated in fibroblasts 2 were enriched in skin barrier and keratinization, and the genes downregulated in fibroblasts 1 are enriched in epidermal development (Fig. 2F). These results indicate that at 7 days after frostbite, there are more fibroblasts to achieve regulation of the nervous system, vascular regeneration, and the immune or ECM microenvironment.

### Skin organoids alleviate the inflammatory response in frostbite model mice

**Figure 3.**
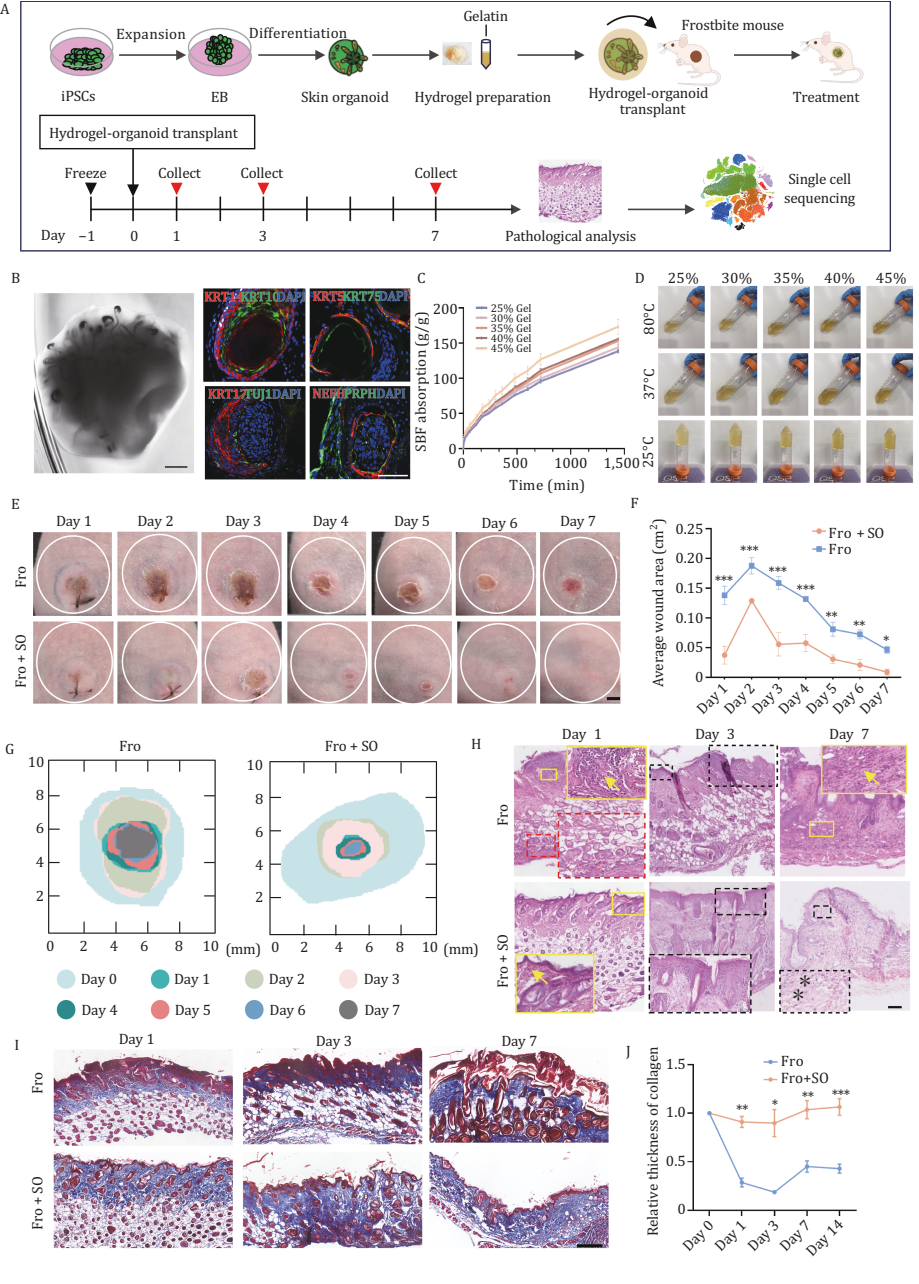
Gelatin-hydrogel wrapped skin organoids promote wound healing in frostbite-affected skin. (A) Schematic representation of the hiPSC-derived skin organoid culture process, gelatin-hydrogel wrapped skin organoids transplantation. A comprehensive flowchart of time points for frostbite model construction, skin organoids treatment, and collecting skin samples for pathological analysis and single-cell sequencing is shown in the lower panel. (B) Bright-field images of skin organoids (scale bar: 500 µm) and immunofluorescence staining of KRT14, KRT10, KRT5, KRT75, KRT17, TUJ1, NEFH, and PRPH (scale bar: 50 µm). (C) Simulated body fluids (SBF) (PBS with 0.5% Tween 80, pH = 7.4) absorbency was calculated using weight change basis. Initially, the weight of solidified hydrogels was measured, and immersed in SBF for 24 h. The weight at different time point of the sample was retaken after surface SBF was removed by gently dabbing with tissue paper. Then SBF absorbency was calculated using equation: SBF absorbency (g/g) = (Mt − Mi)/Mt, where Mt and Mi are the moment and initial weight of the hydrogels. (D) State of the gelatin-hydrogels at 25, 37, and 80°C with different concentrations. The sample solutions formed gels at 25°C for 1 h, which returned to transparent solutions after heating at 37 or 80°C for 15 min, indicating that the hydrogels had reversible temperature-sensitive sol–gel transition properties. (E) Images of frostbite-related wounds of skin tissues in mice treated with (Fro + SO) or without skin organoids (Fro) from day 1 to day 7 (scale bar: 2 mm). The experiment was repeated three times. (F) Line chart of the average wound area in frostbite model mice treated with or without skin organoids (*P < 0.05, **P < 0.01, ***P < 0.001). (G) Schematic representation of the frostbite-related wound area in mice treated with or without skin organoids (scale bar: 2 mm). (H) H&E staining of frostbite-affected skin tissues in mice treated with or without skin organoids (scale bar: 100 µm). On day 1 images, the solid lines indicate inflammatory cells in epidermis with the arrows pointing to the macrophages and the dashed lines show the disrupted blood vessels and red blood cells. On day 3 images, the dashed lines show epidermal structure. On day 7 images, the solid lines indicate inflammatory cells in dermis with the arrows pointing to the macrophages and the dashed lines show dermal structure with the asterisks pointing to the fibroblasts. (I) Masson staining of frostbite skin tissues in mice treated with or without skin organoids (scale bar: 250 µm). The black arrow indicates the collagen thickness. The experiment was repeated three times. (J) Line chart showing the relative thickness of collagen of skin tissues in mice treated with or without skin organoids (*P < 0.05, **P < 0.01, ***P < 0.001). hiPSC: human-induced pluripotent stem cell; EB, embryoid body; Fro: frostbite; Fro + SO: frostbite with skin organoid treatment.

The skin organoids were constructed as in our previous study (Li et al., 2022) (Fig. 3A) and expressed with epidermal cell markers (KRT14, KRT5, and KRT10), hair follicle appendage markers (KRT17, KRT75, and KRT10), and dermal nervous cell markers (TUJ1, NEFH, and PRPH) (Fig. 3B). Then, these skin organoids were fixed by gelatin-hydrogel (Fig. 3C and 3D) and transplanted into frostbite model mice to investigate their therapeutic effect on skin tissues injured by frostbite (Fig. 3A). The results showed that skin organoid transplantation promoted wound healing in frostbite-affected skin (Fig. 3E–G). Pathological analysis revealed significant amelioration of inflammation and vascular impairment (Fig. 3H). Masson staining also showed that skin organoid transplantation promoted rapid recovery of collagen expression in skin tissues affected by frostbite (Fig. 3I and 3J).

**Figure 4.**
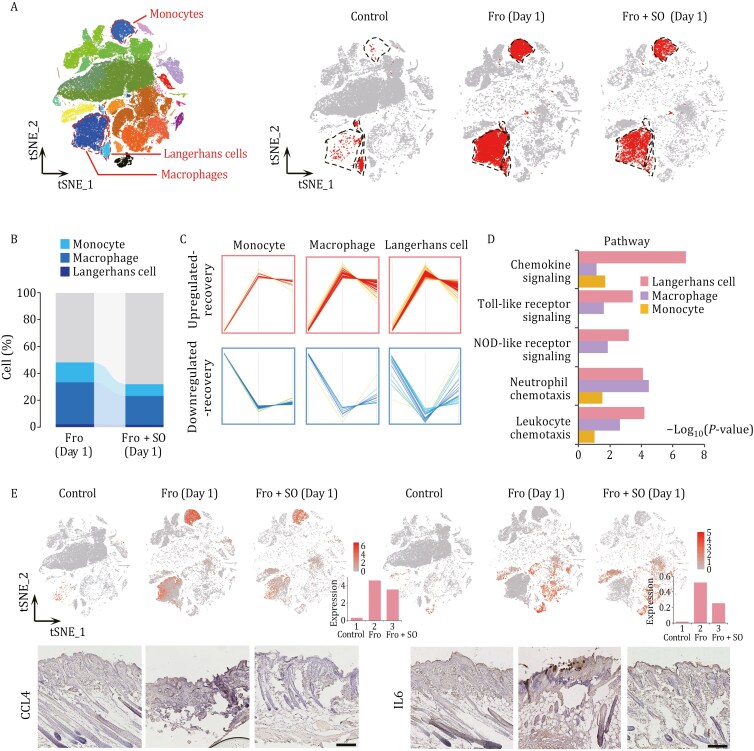
Skin organoids alleviate the inflammatory response in frostbite model mice. (A) Distribution of immune cells (Langerhans cells, macrophages, and monocytes) in skin samples from the control (normal mice skin), 1 day frostbite, and skin organoid-treated 1 day frostbite groups. The dashed lines indicate Langerhans cells, macrophages, and monocytes, respectively. (B) The proportions of Langerhans cells, macrophages, and monocytes in skin samples from frostbite model mice treated with or without skin organoid on day 1. (C) Changes in the expression of genes in Langerhans cells, macrophages, and monocytes that were dysregulated in frostbite and restored to normal levels by skin organoid treatment. The red and blue lines represent DEGs whose expression increased and decreased, respectively, in frostbite-affected skin but recovered to normal levels after treatment with skin organoids. (D) Pathway analysis of the genes upregulated in frostbite model mice and those whose expression recovered to normal levels after skin organoid treatment. (E) scRNA-seq analysis and immunohistochemical staining of *Ccl4* and *Il6* in control skin, skin collected from mice at 1 day after, and skin collected from skin organoid-treated mice at 1 day after frostbite (scale bar: 250 µm). The tSNE plots show the distribution of cells expressing *Ccl4* and *Il6*, while the bar charts display the average expression levels of *Ccl4* and *Il6*. The staining experiment was repeated three times. Fro: frostbite; Fro + SO: frostbite with skin organoid treatment.

Next, we found that the proportions of immune cells including monocytes, macrophages, and Langerhans cells, were decreased in skin tissues with skin organoid transplantation for 1 day compared to those without treatment (Fig. 4A and 4B), indicating that organoid treatment may modulate the immune response in the initial stage of frostbite. To further investigate the functional effects of skin organoids on immune cells in the frostbite model, we compared gene expression between immune cells from model mice at 1 day after frostbite and those from normal mice. There were 104, 875, and 762 upregulated genes and 284, 155, and 252 downregulated genes in monocytes, macrophages, and Langerhans cells from frostbite model mice compared to those from normal mice (Table S2); these genes may be key genes leading to immune cell activation and dysregulation of inflammatory factors after frostbite. Among the DEGs, the expression of 43, 64, and 95 genes was found to be restored to normal levels in monocytes, macrophages, and Langerhans cells, respectively, in the skin of organoid-treated frostbite model mice (Fig. 4C). Biological pathway analysis revealed the genes that were upregulated in monocytes, macrophages and Langerhans cells in skin tissues injured by frostbite but showed recovered expression after skin organoid treatment were associated mainly with chemokine signaling, Toll-like receptor signaling, and NOD-like receptor signaling (Fig. 4D). *Ccl4* and *Il6* are downstream inflammatory factors that activate the Toll-like receptor signaling pathway (Tam et al., 2019). The results of both single-cell transcriptomic analysis and pathological staining showed that CCL4 and IL6 were more highly expressed in skin tissues injured by frostbite than normal skin tissue; moreover, after organoid treatment, the expression of CCL4 and IL6 decreased in skin tissues injured by frostbite (Fig. 4E), suggesting that skin organoid treatment can reduce the inflammatory response in wounds caused by frostbite, possibly preventing an excessive inflammatory response due to repeated activation of inflammatory factors in the injured tissues.

### Skin organoids promote epidermal cell function during tissue repair in frostbite model mice

**Figure 5.**
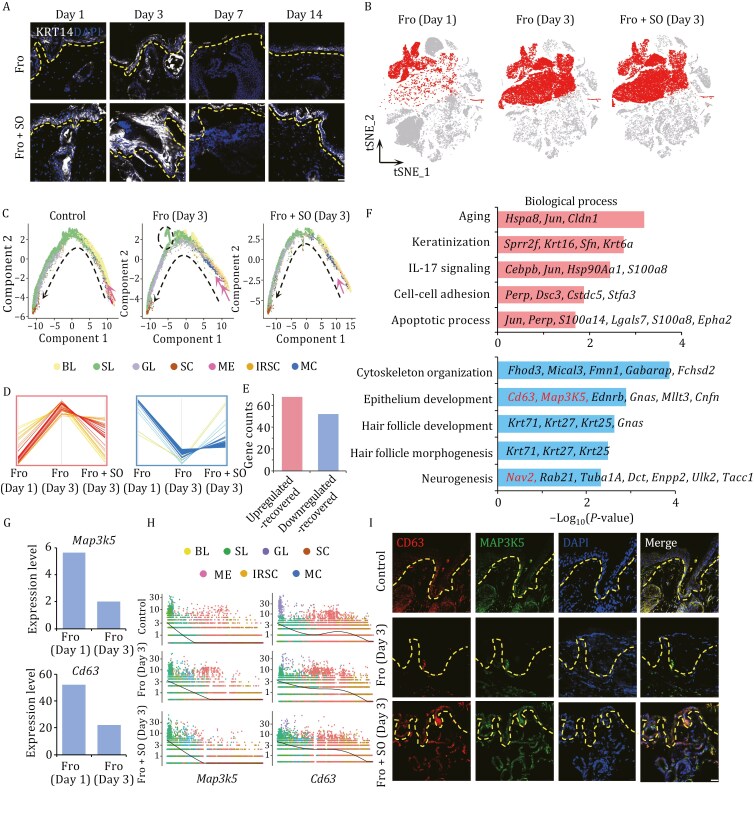
Skin Organoids promote epidermal cell function during tissue repair in frostbite model mice. (A) Immunofluorescence staining of KRT14 in the skin tissues of frostbite model mice treated with or without skin organoids (scale bar: 10 µm). The dashed lines show the basement membrane zone of the skin tissues. The experiment was repeated three times. (B) Distribution of epithelial cells (including basal layer cells, spinous layer cells, granular layer cells, and stratum corneum cells, melanocytes, inner root sheath cells, and medulla cells) in skin samples from frostbite model mice at 1 day and 3 days and those from skin organoid-treated frostbite model mice at 3 days. (C) Single-cell pseudotime trajectories analysis of epithelial cells in skin samples from normal control mice, frostbite model mice at 3 days, and skin organoid-treated frostbite model mice at 3 days. The black dashed lines represent the direction of cell differentiation, while the pink dashed lines indicate the initiation of melanocyte differentiation trajectory. Circular dashed lines in the figure represent the trajectory of basal and inner root sheath cells differentiating abnormally into spinous layer cells. Changes in the expression (D) and distribution (E) of genes whose expression in epithelial cells was dysregulated in the skin of frostbite model mice but recovered to normal levels in the skin of skin organoid treatment frostbite model mice. (F) Biological process analysis of genes whose expression in epithelial cells was upregulated or downregulated in frostbite model mice and recovered to normal levels in skin organoid-treated frostbite model mice. (G) Changes in the expression of *Cd63* and *Map3k5* in frostbite model mice between days 1 and 3. (H) Changes in the expression of *Cd63* and *Map3k5* along the pseudotime trajectory of epithelial cells in the normal control, frostbite, and frostbite and skin organoid treatment groups according to scatter plots with regression curves. The dots of different colors represent different subtypes of epithelial cells. (I) Immunofluorescence staining of CD63 and MAP3K5 in the skin tissues of normal control mice and frostbite model mice treated with or without skin organoids (scale bar: 10 µm). The dashed line shows the basement membrane zone of the skin tissues. The experiment was repeated three times. Fro: frostbite; Fro + SO: frostbite with skin organoid treatment; SL: spinous layer cell; GL: granular layer cell; IRSC: inner root sheath cell; BL: basal layer cell; MC: medulla cell; ME: melanocyte; SC: stratum corneum cell.

Re-epithelialization is an important step in the healing of skin tissues and is mainly achieved by the continuous proliferation and differentiation of epidermal stem cells in the basal layer. The expression of the epidermal stem cell marker KRT14 was increased in skin tissues injured by frostbite at 1, 3, 7, and 14 days after skin organoid transplantation, which was consistent with the previous single-cell transcriptome data (Fig. 5A and 5B), indicating that re-epidermalization of frostbite-affected tissue was enhanced after skin organoid treatment. To further investigate the effect of skin organoid treatment on epidermal cells, we constructed a single-cell trajectory to analyze the differentiation of stem/progenitor cells into epithelial cells in different samples using Monocle 2 method. The results revealed that the cells in the basal layer and some of the inner root sheath cells were at the beginning of the trajectory, the cells in the spinous layer were in the middle of the trajectory, and the cells in the granular layer and stratum corneum were in the terminal state (Fig. 5C). In normal skin tissue, basal stem cells differentiated into the cells of spinous layer, granular layer and stratum corneum cells (Fig. 5C), which is consistent with the findings of previous studies (Leng et al., 2020; Li et al., 2022). However, we found that frostbite interfered with the normally transition order, greatly reducing the number of melanocytes at the beginning of the trajectory and resulting in the irregular differentiation of basal and inner root sheath cells into spinous layer cells (Fig. 5C). After organoid treatment, the number of melanocytes at the beginning of the trajectory begin to recover, and the developmental trajectories of cells in different epidermal layers and hair follicle cells were more similar to those in normal skin tissues (Fig. 5C). These results suggest that skin organoids are capable of treating frostbite by correcting the developmental trajectory of basal stem cells and hair follicle cells.

In previous studies, we found that inflammatory cells were the dominant cells on day 1 after frostbite in skin tissues, while epidermal cells were the dominant cells on day 3 (Fig. 1H and 1I). Therefore, to further investigate the regulatory effect of skin organoids on epithelial cells, we analyzed the DEGs in epidermal cells between days 3 and 1 after frostbite. Among the upregulated and downregulated genes in epidermal cells on days 3 and 1 after frostbite, the expression of 54 and 49 genes was restored to normal levels after skin organoid treatment (Fig. 5D and 5E). The upregulated-restored genes were enriched mainly in aging (*Hspa8*, *Jun*, etc.), keratinization (*Sprr2f*, *Krt16*, etc.), the IL17 signaling pathway (*Cebpb*, *Jun*, etc.), and apoptosis (*Jun*, *Perp*, etc.) (Fig. 5F), indicating that organoid treatment could slow skin epithelial aging, inflammation, and apoptosis. On the other hand, the downregulated-restored genes were enriched mainly in cytoskeleton organization (*Fhod3*, *Mical3*, etc.), epidermal development (*Cd63*, *Map3k5*, etc.), hair follicle development (*Krt71*, *Krt25*, etc.) and nerve regeneration (*Nav2*, *Rab21*, etc.) (Fig. 5F), indicating that skin organoid treatment may aid in the regeneration of hair follicles and nerves while repairing wounds resulting from frostbite.

Further, we found *Map3k5* and *Cd63* were downregulated in basal layer cells in the skin of frostbite model mice at 3 days compared to 1 day (Fig. 5G). *Cd63* plays a role in the activation of ITGB1 and integrin signaling, which is essential for the development and maturation of melanocytes (van Niel et al., 2011). *Map3k5* was found to mediate signaling related to the determination of cell fate, such as differentiation and survival (Sayama et al., 2001). Single-cell transcriptome analysis revealed that *Map3k5* and *Cd63* were highly expressed in the basal layer in the normal and frostbite groups (Fig. 5H). These results indicate that *Cd63* and *Map3k5* may be expressed in epidermal cells in the basal layer, suggesting their potential role in basal stem cells. Immunofluorescence was performed to verify the expression of CD63 and MAP3K5 in the basal layer of the epidermis, and the results showed that CD63 and MAP3k5 expression was decreased after frostbite but increased after skin organoid treatment (Fig. 5I), indicating that organoids may promote re-epidermalization of frostbite-injured skin by regulating gene expression in skin stem/progenitor cells in the basal layer. These results suggest that the rapid increase in the number of epithelial cells on day 3 after skin organoid treatment in frostbite model mice may be an important mechanism of rapid wound repair.

### Skin organoid-mediated regulation of ECM remodeling in fibroblast

Previous studies show that an increase in chemotactic factors and pro-inflammatory factors (such as IL6 and CCL4) can cause to the chronic inflammatory environment, which may lead to abnormal ECM remodeling and subsequent scar formation (Kenny et al., 2023). Fibroblasts are important cell sources of ECM components. To further investigate the gene expression of ECM components in skin fibroblasts at 7 days after frostbite, we identified differentially expressed ECM components on days 7 and 3 after frostbite. We found 110 that the gene expression of six types of ECM components, including 15 collagens, 35 ECM glycoproteins, 7 proteoglycans, 22 ECM regulators, 11 ECM-affiliated proteins, and 20 secreted factors, was upregulated; additionally, the gene expression of 60 ECM components, including 4 collagens, 16 ECM glycoproteins, 2 proteoglycans, 20 ECM regulators, 10 ECM-affiliated proteins, and 8 secreted factors, was downregulated on day 7 compared with day 3 after frostbite (Fig. S5A). Further, fibroblast 5 exhibited the highest number of dysregulation ECM-related genes compared to other fibroblast subtypes (Fig. S5B and S5C), suggesting that fibroblast 5 may serve as a significant source of ECMs during the process of ECM remodeling. Among the ECM components with the highest expression levels in FB5, some are associated with scar formation (including *Col1A1*, *Col3A1*, *Aebp1*, *Fbln2*, *Gldn*, *Igfbp4*, *Mfap4*, *Pcolce*, and *Vcan*), and others are involved in regulating of collagen synthesis, tissue structure, degradation, and ECM remodeling (including *Adamts2*, *Adamtsl1*, *Cst3*, *Loxl2*, *Mmp16*, *Plod2*, *Plod3*, *Serpinf1*, *Serpinh1*, *Sulf1*, *Anxa6*, *Lgals1*, and *Timp3*) (Fig. S5D).

Interestingly, at 7 days after skin organoid treatment, the expression of several upregulated ECMs (*Col1a1*, *Col1a2*, etc.) and downregulated ECMs (*Cilp*, *Cst6*, etc.) returned to almost normal levels in frostbite-affected tissues (Fig. S6A). Immunohistochemical staining results showed that the upregulated ECMs COL1A1 and COL3A1 recovered to the normal levels on mouse skin tissues after organoid treatment on day 7 (Fig. S6B). CLIP, downregulated at 7 days after frostbite, is involved in the synthesis and degradation of ECM, which may have a positive effect on inhibiting scar formation. Some studies reported that the expression level of CST6 is significantly decreased in scar tissues, suggesting that CST6 may be involved in regulating the synthesis and degradation of collagen fibers and thereby affect the extent of scar formation. Additionally, CST6 may inhibit the activity of some proteases and thus affect the biological processes related to scar formation such as cell migration and invasion. We also verified the expression of CLIP and CST6 with downregulated expression in the frostbite group on day 7 compared to day 3 and found that they were upregulated after skin organoid treatment (Fig. S6B). These findings suggest that in the late stage of frostbite, skin organoids treatment may inhibit scar formation by regulating the expression levels of ECM secreted by fibroblasts.

### Skin organoid treatment inhibits the process of fibroblast-to-myofibroblast transition in frostbite mice

**Figure 6.**
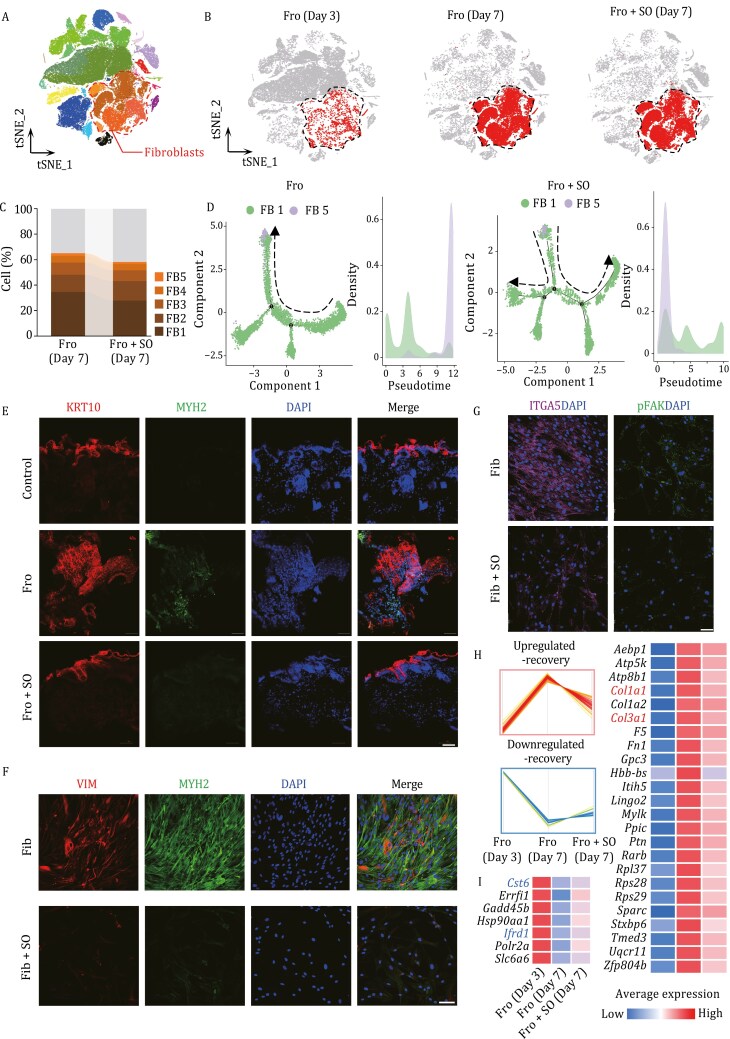
Skin organoids regulate ECM remodeling during tissue repair in the skin of frostbite model mice. (A and B) Distribution of fibroblasts (FB1–5) in skin samples from frostbite model mice at 3 and 7 days and in those from skin organoid-treated frostbite model mice at 7 days. (C) The proportions of different types of fibroblasts in skin samples from frostbite model mice treated with or without skin organoids on day 7. (D) The trajectory analysis of FB1 and FB5 of skin tissues in mice treated with or without skin organoids at 7 days. The direction of dashed lines represents the direction of pseudotime. (E) Immunofluorescence staining of KRT10 and MYH2 in the skin tissues from normal control mice and frostbite model mice treated with or without skin organoids at 7 days (scale bar: 500 µm). The experiment was repeated three times. (F and G) Immunofluorescence staining of VIM, MYH2, ITGA5, and p-FAK in the primary fibroblasts before and after co-culture with skin organoids (scale bar: 500 µm). The experiment was repeated three times. (H) Expression changes of genes that were dysregulated in fibroblasts of frostbite model mice skin but recovered to normal levels in the skin of frostbite model mice treated with skin organoids. (I) Heatmap showing the upregulation and downregulation trends of genes of FB5 presented in (H). The boxes with different colors indicate the normalized average values of the upregulated and downregulated expression levels of genes in FB5, respectively. Fro: frostbite, Fro + SO: frostbite with skin organoid treatment, FB1: fibroblast 1, FB2: fibroblast 2, FB3: fibroblast 3, FB4: fibroblast 4, FB5: fibroblast 5, Fib: human-derived fibroblasts, Fib + SO: human-derived fibroblasts co-cultured with skin organoids.

To further investigate the mechanisms by which organoids impact the number of fibroblasts to regulate ECM levels during the late stage of wound healing, we analyzed changes in fibroblasts at the single-cell level. Results showed that at 7 days after frostbite, there is an overall increase in fibroblast numbers in the skin tissue (Figs. 1J, 6A, and 6B); however, the proportions of fibroblasts decrease after skin organoids treatment. Furthermore, we observed a decrease in the proportions of fibroblast 1 and fibroblast 5 among the 5 fibroblast subtypes following treatment with skin organoids (Fig. 6C). Given previous findings suggesting the potential involvement of fibroblast 5 in scar formation, we conducted further analysis and identified that fibroblast 5 exhibits higher expression levels of Hhip and Vim compared to other fibroblasts (Fig. 1G), indicating its potential as myofibroblasts.

Myofibroblasts play a pivotal role in tissue repair and scar formation processes (Oishi et al., 2024). They exhibit various characteristics, including contractile ability resembling that of muscle cells and expression of α-smooth muscle actin (Younesi et al., 2024). Myofibroblasts primarily appear during the repair of damaged tissues, particularly in the process of scar formation. The main functions of myofibroblasts include synthesizing and secreting collagen, thereby promoting tissue repair, but they are also associated with abnormal scar formation, such as hypertrophic scarring and fibrosis (Ackerman et al., 2024; Younesi et al., 2024). Their excessive activity within scar tissue can lead to overformation and contraction of scar tissue, resulting in functional impairments and unfavorable cosmetic outcomes.

**Figure 7.**
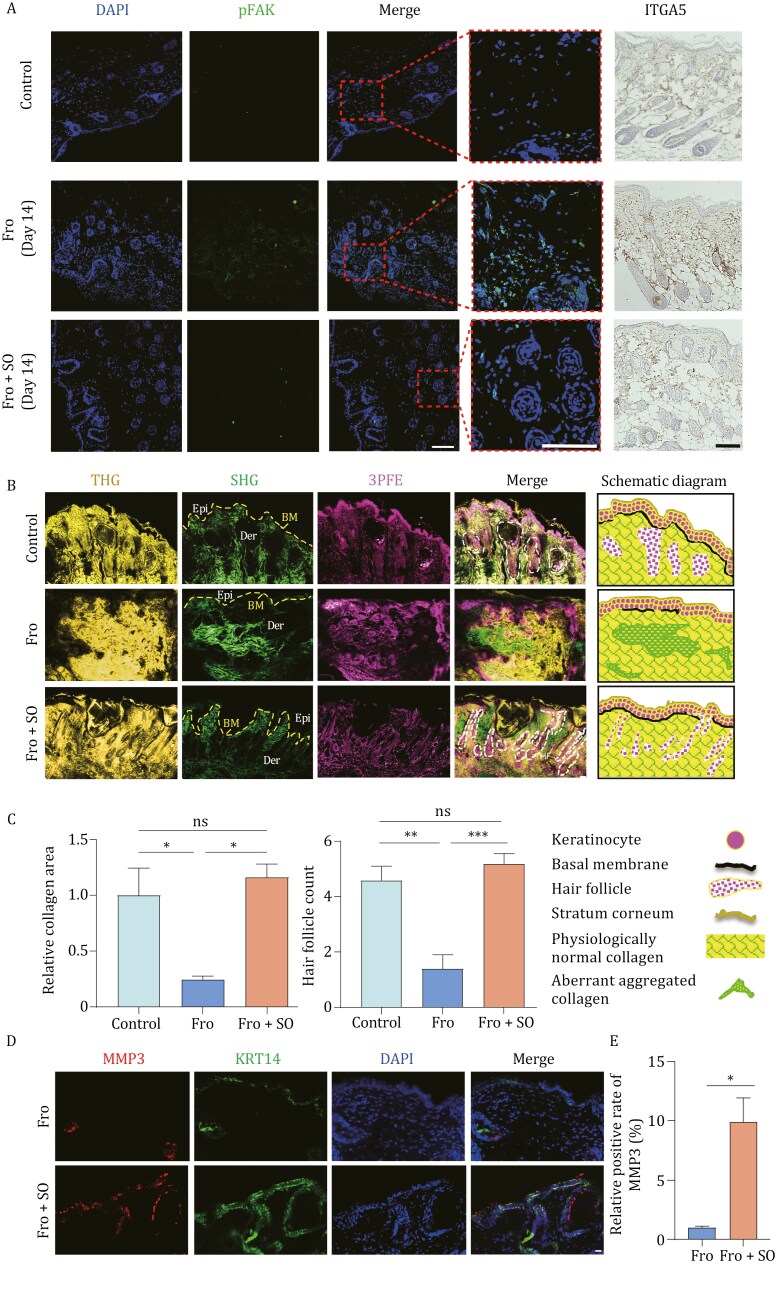
Skin organoids reduce the formation of long-term scars in frostbite mouse models. (A) Immunofluorescence staining of p-FAK and immunohistochemical staining of ITGA5 in mouse skin with and without skin organoid treatment at 14 days after frostbite, and in normal controls (scale bar: 100 µm). The experiment was repeated three times. (B) Images analysis of cell morphology (third harmonic generation: THG), energy metabolism (three-photon excitation fluorescence: 3PFE), and collagen fibers (second harmonic generation: SHG) in the normal mouse skin, mouse skin after 14 days of frostbite, and mouse skin after skin organoid treatment for frostbite, captured by femtosecond multiphoton  microscopy  (scale bar: 500 µm). The dashed lines represent the basement membrane of the skin, and the white dashed box outlines the area of the hair follicle. The experiment was repeated three times. (C) Bar chart depicting the relative collagen area and hair follicle count of normal mouse skin, mouse skin after 14 days of frostbite, and mouse skin after skin organoid treatment (*P < 0.05, **P < 0.01, ***P < 0.001). (D) Immunofluorescent staining of MMP3 and KRT14 in the skin tissues from Fro and Fro + SO at 7 days after frostbite injury (scale bar: 500 µm). The experiment was repeated three times. (E) Bar chart of relative positive rate of MMP3 (*P < 0.05, **P < 0.01, ***P < 0.001).

Immunofluorescence staining showed that the expression levels of myofibroblast marker (MYH2) increased in the frostbite group, however decreased after skin organoids treatment compared to the normal group (Fig. 6E), which further demonstrates that the treatment of skin organoids for frostbite can reduce the number of myofibroblasts. Furthermore, through pseudotime analysis, we find that fibroblast 1 is transitioning towards fibroblast 5 (myofibroblasts), and myofibroblasts disappear earlier after organoid treatment, compared with that without treatment (Fig. 6D). This suggests that the treatment with skin organoids may reduce the number of myofibroblasts at 7 days after frostbite by regulating the transition of fibroblasts to myofibroblasts. Compared to human-derived fibroblasts, co-cultured human-derived fibroblasts with skin organoids exhibited lower expression of myofibroblast markers (VIM and MYH2) (Fig. 6E and 6F), and immunofluorescence staining results for mechanical force-related proteins p-FAK and integrin-α5 (ITGA5) show significantly higher expression levels in the frostbite group compared to the treatment group (Fig. 6G), indicating that skin organoids can inhibit the integrin α5β1-FAK pathway to reduce excessive fibroblast-to-myofibroblast transitions. Similarly, in the mice skin tissues, the expression levels of ITGA5 and p-FAK were significantly higher in the dermis of the untreated group compared to the normal controls, and their expressions recovered to normal levels in the skin organoids treated group (Fig. 7A). These results at both the cellular and tissue levels indicate that skin organoids can inhibit the integrin α5β1-FAK pathway, thereby reducing excessive transitions from fibroblasts to myofibroblasts.

### Skin organoid suppresses scar formation in frostbite

Furthermore, we found that after treatment with skin organoids, some downregulated genes in fibroblasts 5 (myofibroblasts) (*Cst6*, *Ifrd1*, etc.) and upregulated genes to normal levels, while the upregulated genes (*Col1a1*, *Col3a1*, etc.) were restored to normal levels (Fig. 6H). Among them, *Ifrd1* may participate in cell proliferation and signaling transduction, promoting the formation of scar tissue. *Cst6* may be involved in regulating protease activity, impacting the formation of scar tissue and the balance of ECM degradation. *Col1a1* and *Col3a1*, two major components of scar tissue, play significant roles in the process of scar formation. These results indicate that skin organoids can regulate the gene expression of myofibroblasts to secrete ECM, which may be a contributing factor to scar formation.

In addition, myofibroblasts can secrete large amounts of collagen. At 14 days after frostbite, there is abnormal aggregation of collagen in the dermis, disrupted distribution, and compromised integrity of the basement membrane, compared to the normal group (Fig. 7B), which promotes scar formation after frostbite. After treatment with the skin organoids, there was a decrease in collagen content, and its distribution pattern resembled that of the normal control group. Additionally, normal hair follicle structures disappeared, and quantification of NAD(P)H and FAD fluorescence shows elevated oxidative phosphorylation and glucose metabolism levels in skin tissue at day 14 after frostbite (Fig. 7B and 7C). The changes in follicular structure may impact cellular behaviors during skin regeneration and repair processes, thereby influencing scar formation. Additionally, energy metabolism plays a crucial role in cellular functions and biological processes such as cell proliferation, migration, and matrix synthesis. Hence, abnormalities in energy metabolism may affect cellular activity and tissue repair capabilities during scar formation. Specifically, damage or loss of follicular structure may lead to aberrant fibrotic reactions during skin repair, thereby promoting scar formation. Conversely, disturbances in energy metabolism may impair cellular activity, affecting the formation and repair capabilities of scar tissue. Furthermore, follicular structure and energy metabolism may also modulate scar formation processes through the regulation of cellular signaling pathways, gene expression, and protein synthesis. Matrix Metalloproteinases (MMPs) facilitated the degradation and remodeling of scar tissue by aiding in the clearance of excessive deposits of collagen and other ECM components, thereby promoting scar repair and remodeling. An increase in MMP3 levels is detected after skin organoid treatment, indicating the inhibition of scar formation (Fig. 7D and 7E). These findings demonstrate that skin organoids regulate scar formation by controlling ECM expression, collagen arrangement, energy metabolism, and ECM enzyme systems in dermal fibroblasts, with the aim of suppressing scar formation.

## Discussion

In recent years, many places have experienced unprecedentedly cold winters due to global climate change, and the incidence of frostbite among civilians living in harsh winter conditions and at subzero temperatures is increasing. Therefore, we believe that it is important to further explore the pathophysiology of frostbite and to develop appropriate treatments. However, the patterns of cellular and molecular changes during the process of frostbite are not clear, as frostbite has been described only as a thrombotic ischemic condition (Joshi et al., 2020). Frostbite can be accompanied by many complications, such as infection, chilblains, scarring, chronic paresthesia, chronic pain, and hyperhidrosis. These problems are largely due to delayed wound healing and the inability of nerves and blood vessels in skin and skin appendages to regenerate. Long-term complications are common even in healthy individuals with grade one and two frostbite (Regli et al., 2021). However, the current treatment methods for frostbite are limited and cannot prevent the development of complications. At present, there have been few studies on frostbite treatment, and related reports are mainly retrospective analyses, literature reviews, and expert opinions (Jin et al., 2021; Sheridan et al., 2022). Here, we developed a grade three frostbite mouse model using a freeze–thaw–freeze cycle and evaluated the pathological characteristics and changes in different cell types after frostbite. Excitingly, we found that skin organoids accelerated the healing of wounds resulting from frostbite and reduced scar formation in the later stages of recovery.

**Figure 8.**
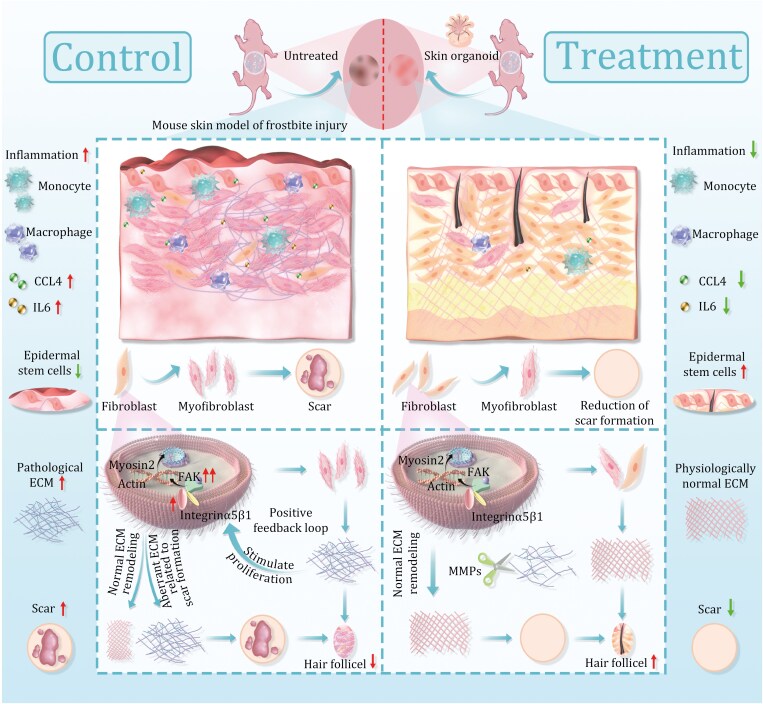
Mechanism diagram illustrating of skin organoid treatment reduces scar formation in frostbite skin tissues. The skin organoids for treating frostbite-damaged skin can reduce inflammatory cells and cytokines, and restore the number and normal arrangement of epidermal stem cells to accelerate wound healing. Importantly, skin organoids reduce the overall number of fibroblasts, significantly attenuate fibroblast-to-myofibroblast transition by modulating the integrin α5β1-FAK pathway, and remodel the ECM through degradation and reassembly mechanisms. This facilitates the restoration of physiological ECM and reduces the abundance of ECM associated with abnormal scar formation, thereby aiding in scar reduction and preventing follicular damage. Fro: frostbite; Fro + SO: frostbite with skin organoid treatment; Epi: epidermis; Der: dermis; BM: basement membrane.

The healing of frostbite-induced injury is broadly consistent with the general skin healing process and includes three stages: inflammation, proliferation, and maturation (Broughton et al., 2006). Compared with common skin wounds, frostbite-related wounds are associated with a more robust ischemic response, more severe nerve necrosis, and massive accumulation of mononuclear macrophages rather than neutrophils at 1 day after frostbite. This may be because frostbite-related wounds are relatively sterile, weakening the function of neutrophils. Monocytes are precursors of macrophages, and during injury, monocytes are recruited to skin tissue and differentiate into macrophages according to the cues provided by the damaged skin microenvironment. We found that monocytes and macrophages are the two major inflammatory cell types involved in the initial inflammatory phase after frostbite. In macrophages, genes responsible for regulating tumor necrosis factor, IFN-γ, and IFN-β expression, which can drive the initial cellular inflammatory response after skin injury, are upregulated. Indeed, the expression of genes associated with T-cell, IL4, and IL2 was downregulated in macrophages during the initial phase of frostbite, indicating that acquired immunity does not play a major role in this phase. In addition, the number of Langerhans cells changed only slightly, but these cells produced the largest amount of chemokines; moreover, genes associated with the skin barrier and keratinization were downregulated in Langerhans cells, indicating that the ability of these cells to remove invasive bacteria and thus help protect the body from various stimuli and promote repair after injury is weakened in the initial phase of frostbite. We used our previously established skin organoids containing epidermis, dermis, neurons, and hair follicle cells to treat skin injury caused by frostbite. Surprisingly, skin organoids quickly promoted the healing of wounds caused by frostbite, even at 1 day after treatment. We also found that skin organoids decreased the proliferation of inflammatory macrophages, thereby reducing the levels of inflammatory cytokines. This is important because a highly inflammatory state hinders the transition to the next phase of frostbite, and prolonged inflammation can lead to the continuous secretion of ECM components by fibroblasts or myofibroblasts, which leads to physiological or pathological scar formation (Wang et al., 2020). Thus, skin organoids may be able to inhibit the formation of scars after frostbite (Fig. 8).

After injury, it is essential to trigger a wound-healing response to achieve rapid repair of the epidermis and restoration of the skin barrier, and a delay in the re-epithelialization process leads to a greater incidence of infection and chronic wound formation. Under homeostatic conditions, the differentiation trajectory of each stem cell population is determined by the microenvironment, and when basal cells exit the basal layer, they stop proliferating and begin to differentiate, forming the spinous, granular, and stratum corneum layers (Gonzales and Fuchs, 2017). However, after injury, these stem cell populations exhibit significant plasticity when the local environment changes significantly. We found that the proliferation of epidermal stem cells was disrupted during frostbite, possibly resulting from the stimulation of keratinocytes by inflammatory factors secreted by inflammatory cells. Three days after frostbite, the number of keratinocytes increased significantly in preparation for epidermal regeneration. Excitingly, we observed successful epidermal regeneration at 3 days after treatment. Moreover, the number of keratinocytes was normal, but the number of epidermal stem cells was increased, and they were functional. This suggests that skin organoids improved the function of epidermal stem cells while promoting their proliferation. Next, we found that a very large number of genes were downregulated in different kinds of epidermal cells at 3 days after frostbite; these genes may be the key genes responsible for the dysregulation of epidermal stem cells. Surprisingly, the expression of these downregulated genes increased after treatment with skin organoids. We found that some of these genes, such as *Cd63* and *Map3k5*, are mainly associated with epidermis and hair follicle development and are specifically expressed in epithelial progenitors in the basal layer. These findings suggest that skin organoids may promote re-epithelialization of skin after frostbite by regulating gene expression in epithelial progenitors.

The freezing injury can affect the mechanical properties of cells. When tissues suffer from low-temperature damage, the cells face various stresses, including mechanical stresses during the freezing and thawing processes, which may lead to changes in the mechanical properties of cells. During frostbite, cells may undergo mechanical compression during ice crystal formation and osmotic pressure differences due to an imbalance in solute concentration inside and outside the cells during thawing, both of which can impact the morphology and structure of cells. Since the fibroblasts are able to contract and have a close relationship with the ECM, they are highly sensitive to mechanical environment. We found that 7 days after frostbite, the integrin α5β1FAK pathway in fibroblast cells is activated. The integrin α5β1-FAK pathway is a key mechanical signaling pathway involved in cell adhesion, migration, and communication with the ECM. Integrins are cell surface receptors that mediate cell adhesion to the ECM, with α5β1 being a specific type of integrin. FAK (focal adhesion kinase) is a cytoplasmic protein tyrosine kinase activated in response to cell adhesion mediated by integrins. When cells adhere to the ECM through integrin α5β1, this interaction triggers FAK activation. This activation leads to phosphorylation of FAK on specific tyrosine residues, forming a signaling complex that regulates various cellular processes. Activated FAK can initiate downstream signaling pathways, and in our study, we observed activation of downstream myosin via immunofluorescence staining, transmitting mechanical signals to the nucleus, ultimately activating fibroblasts into myofibroblasts. Excessive myofibroblasts are a major mechanism leading to excessive scar formation. Abnormal activation of myofibroblasts can disrupt ECM generation, promote local scar formation, hair follicle disappearance, and loss of basement membrane integrity. This abnormal distribution and morphology of ECM (*Aebp1* and *Fbln2*) also promote excessive proliferation of myofibroblasts, forming a positive feedback loop. Additionally, inflammatory cells secrete IL6 and CCL4, stimulating fibroblasts to transform into myofibroblasts, increasing the likelihood of scar formation. In summary, the excessive accumulation of inflammatory cells and increased number of myofibroblasts after frostbite are important factors leading to irreversible scar formation in the skin.

Blocking mechanisms related to the mechanical environment and altering the phenotype of myofibroblast cells to obtain cells capable of reshaping abnormal ECM deposition is undoubtedly a novel approach to developing treatments for regulating scar formation. Excitingly, 7 days after skin organoids treatment, there was a significant reduction in myofibroblast quantity, and after 14 days of skin organoids treatment, the number of hair follicles returned to normal, energy metabolism around hair follicles showed normal levels, collagen in the dermis arranged normally, and the basement membrane was intact. This effect was mediated by inhibiting the integrin α5β1-FAK pathway. Through immunofluorescence staining, we found that MMP3 significantly increased after skin organoids treatment for seven days. MMP3 is a metalloproteinase that plays an important regulatory role in the skin. MMP3 can degrade collagen, promote the degradation and clearance of aged or damaged collagen, participate in regulating the composition and structure of the ECM, and influence processes such as cell–cell interactions, cell migration, and signal transduction. This may affect the stiffness and morphology of the matrix surrounding cells, thereby influencing the activation and response of intracellular signaling pathways. Therefore, the elevated levels of MMP3 in the skin after skin organoids treatment may be one of the reasons for the skin organoids affecting the mechanical signaling pathway. However, it is currently unclear how skin organoids inhibit proteins related to the mechanical signaling pathway. Further exploration is needed in the future to investigate the effects of skin organoids themselves or their exosomes on the mechanical signaling pathway.

It is worth noting that in the process of skin organoids transplantation, we used gelatin-hydrogel to wrap the organoids for transplantation. Gelatin is a natural polymer and many studies have confirmed its fascinating biological properties, such as biocompatibility (Li et al., 2023b), biodegradability (Baumgartner et al., 2020), bioactivity (Kim et al., 2021), and its affinity for chondrocyte cells (Murphy et al., 2020). This is also our first attempt to use gelatin-hydrogel to fix the skin organoids to the wound so that the skin organoids can be firmly positioned on the wound. The gelatin will degrade with the combined effect of time and 37°C body temperature, exposing skin organoids out of gelatin-hydrogel for treatment.

A limitation of this study is that BALB/c nude mice lack mature thymus and T lymphocytes, although they do retain some functional immune components, including B cells and innate immune cells (Flanagan, 1966; Morikawa et al., 1988; Tani et al., 1995). However, there are certain advantages to using nude mice as animal models. Due to their immunodeficiency, the nude mice are less likely to cause the immune rejection in xenotransplantation, making they could be a suitable model to study skin organoid transplantation for frostbite treatment. Further research is essential to advance the research results for clinical application, ensuring that patients with frostbite can benefit from this treatment.

In summary, these studies reveal that due to their unique functions, skin organoids could be exploited therapeutically to modulate inflammation as well as fibrosis in refractory wounds caused by skin diseases or in patients with wound-healing defects. Indeed, in a previous study (Ma et al., 2022a), we reported that the application of hiPSC-derived epithelial and mesenchymal organoids to scleroderma-affected skin significantly reduced the degree of skin fibrosis as well as inflammation, promoted the regeneration of the epidermis and skin appendages and reduced inflammation and scarring. These studies all highlight the great potential of skin organoids to repair tissue damaged by different conditions in the foreseeable future.

## Supplementary data

Supplementary data is available at *Protein & Cell* online at https://doi.org/10.1093/procel/pwae055.

pwae055_suppl_Supplementary_Materials

pwae055_suppl_Supplementary_Table_S1

pwae055_suppl_Supplementary_Table_S2

pwae055_suppl_Supplementary_Table_S3

pwae055_suppl_Supplementary_Table_S4

pwae055_suppl_Supplementary_Table_S5

pwae055_suppl_Supplementary_Table_S6

## Data Availability

All scRNA-seq data have been deposited to NCBI with the identifier PRJNA1099380. The equipment, reagents, and supplies are available in Table S6.

## Code availability

No specific custom script was used in this study and codes will be accessed with reasonable requests.
