# Correction to: Skin organoid transplantation promotes tissue repair with scarless in frostbite

**DOI:** 10.1093/procel/pwag001

**Published:** 2026-06-03

This is a correction to: Wenwen Wang, Pu Liu, Wendi Zhu, Tianwei Li, Ying Wang, Yujie Wang, Jun Li, Jie Ma, Ling Leng, Skin organoid transplantation promotes tissue repair with scarless in frostbite, *Protein & Cell*, Volume 16, Issue 4, April 2025, Pages 239–258, https://doi.org/10.1093/procel/pwae055

The legend for Fig. 3J contains an inaccuracy: the **blue line** should represent “**Fro**” (not “Fro + SO”) and the **orange line** should represent “**Fro + SO**” (not “Fro”).

Figure 3J should read:


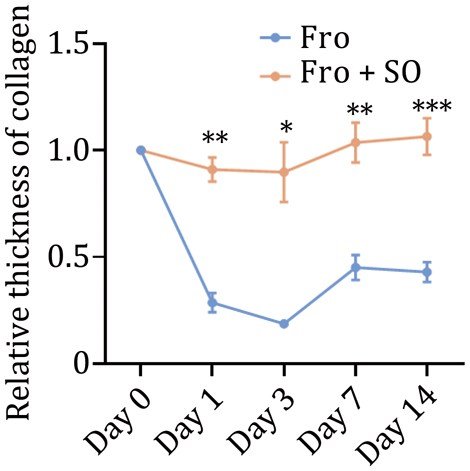


instead of:


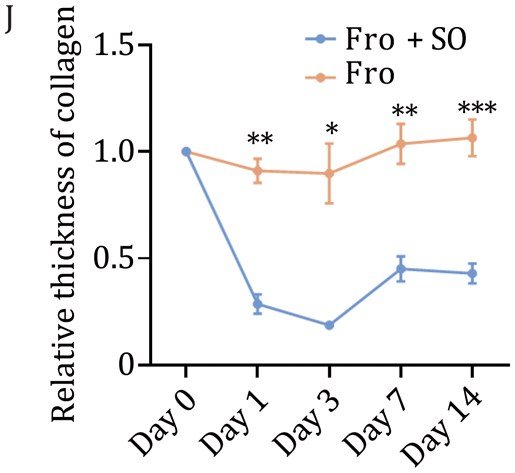


This error does not affect the study’s conclusions or data integrity.

