# Supplementary material for: Skin organoid transplantation promotes tissue repair with scarless in frostbite
Source: Protein Cell. 2024 Oct 4;16(4):239–58. doi: 10.1093/procel/pwae055 (PMC12053479; doi:10.1093/procel/pwae055)
Supplement: pwae055_suppl_Supplementary_Materials [file pwae055_suppl_supplementary_materials.pdf]

Materials and Methods

hiPSC-derived skin organoid culture

The hiPSC-derived skin organoids were produced as described in a previous study (Lee et al., 2022). In brief, hiPSCs were dissociated into single cells and seeded into a low-attachment 96-well U-bottom plate at 3,500 cells/100 µl per well. Then, Essential 8 + 20 µM Y27632 were added to induce the formation of aggregates. After 24 h, 100 µl of Essential 8 was added to each well. The cells were incubated for an additional day, and the aggregates were transferred to new 96-well plates containing 100 µl of Essential 6, 2% Matrigel, 10 nm/ml hFGE and 2.5 nm/ml BMP4 for 3 days. Subsequently, the aggregates were treated with 25 µl of Essential 6. The cells were incubated with 250 ng/mL bFGF and 1 µM LDN for another 3 days. On day 6, 75 µl of fresh Essential 6 was added to provide nutrients, and half of the medium was replaced with fresh Essential 6 every 2 days until day 12. On day 12, the aggregates were transferred to 500 µl OMM containing 24.5 ml Advanced DMEM-F12, 24.5 ml neurobasal medium, 500 µl B27 without vitamin A, 500 µl GlutaMAX, 250 µl N2 supplement, 91 µl 2-mercaptoethanol and 100 µl Normocin (containing 1% Matrigel) and shaken at 65 rpm. On day 15, the half of the medium was replaced with fresh OMM and 1% Matrigel. Half of the medium was replaced with fresh OMM every 3 days from day 18 to organoid maturity.

Animal model of full-thickness wounds caused by frostbite

All animal experiments were performed following established standards with the approval of the Institutional Animal Care and Use Committee at the National Center for Protein Sciences (Beijing) (approval No: NCPSB-20230419-23MO). A total of 21 BALB/c nude male mice were purchased from Beijing Vital River Laboratory Animal Technology Co., Ltd. (Beijing, China). A frostbite mouse model was established according to previous studies (Auerbach et al., 2013). Briefly, nude mice (7 weeks old, weighing 22-25 g, male) were anesthetized and placed on a warming blanket. Before freezing, a 0.8 cm diameter circle was drawn on each nude mouse with a marker to ensure that the frostbite area was standardized. After cooling homemade copper sheets on dry ice, the skin on the back of the nude mice was gently lifted and fully contacted with the cold copper sheet for 1 minute, after which the copper sheet was removed and the skin was allowed to undergo thawing for 3 minutes. After 3 min of thawing, the cold copper sheet was reapplied at the same location. Then, 2 additional freezing cycles were performed. The nude mice were treated with buprenorphine (0.05 mg/kg). After freezing, no additional dressing was applied to the wound.

Preparation of hydrogels and administration of skin organoids to frostbite model mice

Gelatin (3.75, 4.5, 5.25, 6.00, 6.75 g) [Cat#V900863, Sigma] was dissolved in 15 mL PBS using a water bath at 80°C. After cooling to room temperature, hydrogels were prepared and used to wrap the skin organoids. The pathological changes in the skin of the nude mice after the freeze-thaw-freeze method were examined by histological analysis of the frostbite-affected skin. Anesthetized mice were placed on a preheated electric blanket. Then, a 0.5 cm incision was made 0.5 cm lateral to the edge of the frostbite area in each mouse. A single mature skin organoid wrapped by gelatin-hydrogel was transplanted in each incision, after which the wound was closed with a suture. In the control group, a same incision was made in the same location; however, no organoid was transplanted, and the skin was immediately sutured. The skin tissues of frostbite model mice were collected 1, 3, and 7 days after surgery for pathological staining and single-cell sequencing.

Analysis of wound surface photos

Digital photographs of the wounds were taken daily after treatment with skin organoids until the wounds were completely healed according to macroscopic examination. The complete healing time was defined as the time from initial freezing until the wound was fully filled with new tissue. The wound surface area was determined by tracing the wound edge with a computer mouse at a fine resolution. The number of pixels in the wound area was calculated using ImageJ (version 1.38) software. During all measurements, the mice were anesthetized and placed in a relaxed position to mitigate back curvature and ensure consistent anatomy.

Coculture of human dermal fibroblasts with skin organoids

The fibroblasts were cultured using previously described methods. Briefly, after digestion with 2 mg/mL Dispase II at 37 °C for 1 h, the dermis was separated from the epidermal sheets and digested with prewarmed (37 °C) 0.25% trypsin/EDTA for 15 min. The trypsin digestion was neutralized with fetal bovine serum (FBS), and the dermal cells were washed twice with cold PBS to remove trypsin, filtered through a 40-μm strainer, and centrifuged at 1200 rpm for 5 min. For monolayer cultures, the cells were seeded onto plates. DMEM/F-12 medium supplemented with 10% FBS and 1× penicillin/streptomycin (10 μL/mL) was added. After 24 h, the culture medium in the wells was replaced with fresh medium. Mature skin organoids were selected and cocultured with fibroblasts using Advanced DMEM/F-12 medium supplemented with 1% NEAA, 2% B-27, 1% GlutaMAX, 1% HEPES, 1.25 mM N-acetylcysteine, 50 ng/mL hEGF, 50 ng/mL Wnt3a, 1 μM A83, 10 μM Forskolin, and 1× penicillin/streptomycin (10 μL/mL).

Histological and Immunofluorescence Staining

The samples were fixed overnight in 4% formaldehyde at 4°C and dehydrated in graded ethanol solutions. After paraffin embedding, the tissues were cut into 4 μm thick sections for H&E staining, Masson staining, immunohistochemistry, and immunofluorescence. For immunofluorescence staining, the sections were deparaffinized and boiled in antigen retrieval buffer in a microwave for at least 12 min, and endogenous peroxidase activity was blocked with 0.3% hydrogen peroxide for 30 min after cooling. The sections were then blocked with normal horse serum in Tris-buffered saline for 1 h, incubated with reagents from an avidin/biotin blocking kit, and incubated with an antibody overnight at 4°C. The sections were then incubated with secondary antibodies. A Masson three-color staining kit (Solebo. G1340-750 ml) was used for Masson staining.

The cells were fixed in 4% formaldehyde for 20 minutes and then washed with PBS. Subsequently, the cells were treated with 0.25% Triton X-100 for 20 minutes to remove plasma membranes, blocked in 10% serum for 1 hour at room temperature (RT), and incubated with primary antibodies overnight at 4 °C. After a 1-hour incubation at RT with secondary antibodies and counterstaining with DAPI, the sections were sealed with Fluoro-Gel for photography. Negative control samples were incubated with secondary antibodies alone. Images were captured at 20×/40× magnification and analyzed using Volocity Demo (×64).

Multiphoton microscopy imaging

The paraffin-embedded tissue sections were deparaffinized, and a coverslip was placed on the tissue. The section was inverted onto a moving stage and imaged using a femtosecond label-free imaging (FLI) microscope system (Femtosecond Research Center, Guangzhou, China). Backward-scattered signals were separated into multiple channels (THG 340-380 nm; 3PEF 415-526 nm; SHG 530-570 nm, etc.). A non-scanning detection scheme was implemented using a set of dichroic mirrors and bandpass filters (Semrock, Inc.) with a 50 mm focal length lens to focus, and images

from individual channels were finally merged into a composite image with pseudocolors (THG-yellow, 3PEF-green; SHG-green).

Mouse skin cell collection, scRNA-seq, and scRNA-seq data analysis

Skin tissue samples from mice with frostbite injury and those treated with skin organoids were collected on different days to generate single-cell gene expression libraries. Before tissue collection, the mice were euthanized by CO2 inhalation. Skin samples from normal mice were taken as the control group tissues (day 0, n = 3); those from frostbite model mice were taken at 1, 3, and 7 days after frostbite injury as the model group tissues; and those from skin organoid-treated frostbite model mice were taken at 1, 3, and 7 days after frostbite injury and skin organoid treatment as the treatment group tissues (n = 3 for each group).

Then, the mouse skin samples were digested with 0.25% trypsin/EDTA until the samples dissociated into a single-cell suspension with no obvious cell aggregation. The suspension was filtered through a 40 μm Flowmi cell strainer to remove the cell debris; cell umber and viability were estimated using a fluorescence cell analyzer (Countstar® Rigel S2) and acridine orange/propidium iodide to ensure that the cell concentration and viability were suitable for sequencing. Finally, single-cell RNA-Seq libraries were prepared using a SeekOne® MM Single-cell 3' library preparation kit (SeekGene Catalog No. K00104) and subsequently sequenced on an Illumina NovaSeq 6000 with a PE100 read length.

Cell Ranger software (10× Genomics) (https://support.10xgenomics.com/v6.1.1) was used to process the raw scRNA-seq data. The FASTQ files were aligned to the mm10 mouse reference genome to generate a gene expression matrix. The R package Seurat (version 4.1.0) was used for further data analysis. First, low-quality cells were excluded if they met the following criteria: nUMI < 500, nGene < 250, percent_mito> 15%, and log10 GenesPerUMI<0.7. After quality control, a total of 148,389 cells remained. After excluding unnecessary cells, the "NormalizedData" function was used to log-normalize the count, and the top 2000 highly variable genes were determined using the "FindVariableFeatures" function for downstream bioinformatics analyses. Next, we applied the linear transformation "ScaleData" function. The batch effects among samples were removed using the "RunHarmony" function.

Statistical and bioinformatics analysis

Statistical analyses were performed using the R (version 4.0.3). DEGs between the two groups of cells were identified from the scRNA-seq data using the Wilcoxon rank-sum test. Differences for which the Benjamini–Hochberg adjusted p value was less than 0.05 were considered statistically significant. The "FindMarkers" function was used to identify upregulated and downregulated genes between any two sets of samples. Genes with a |logfold change| > 0.25 and adjusted p < 0.05 were identified as DEGs. Single-cell pseudotime trajectories were generated by using Monocle2.

For other analyses, each experiment was performed at least three times (n ≥ 3), and the statistical data are shown as the means ± standard deviations (SD). Statistical tests were conducted using GraphPad Prism (version 8.0). Student's *t* test was performed to compare data between groups. The results were considered significant at $p \leq 0.05$ (*), 0.01 (**), or 0.001 (***), and the specific *p* value is provided in the figure legends.

The online tool DAVID (Sherman et al., 2022) was used to annotate the proteins according to biological processes via Gene Ontology (Ashburner et al., 2000) analysis, and the biological pathways in which the DEGs were enriched were identified using Kyoto Encyclopedia of Genes and Genomes (Ogata et al., 1999) and WikiPathway. The Matrisome database (Naba et al., 2016) was used to annotate the ECM components.

The circlize package (version 0.4.15) (Gu et al., 2014) was used to visualize upregulated and downregulated DEGs involved in multiple biological processes associated with different cell types.

Supplementary Figures

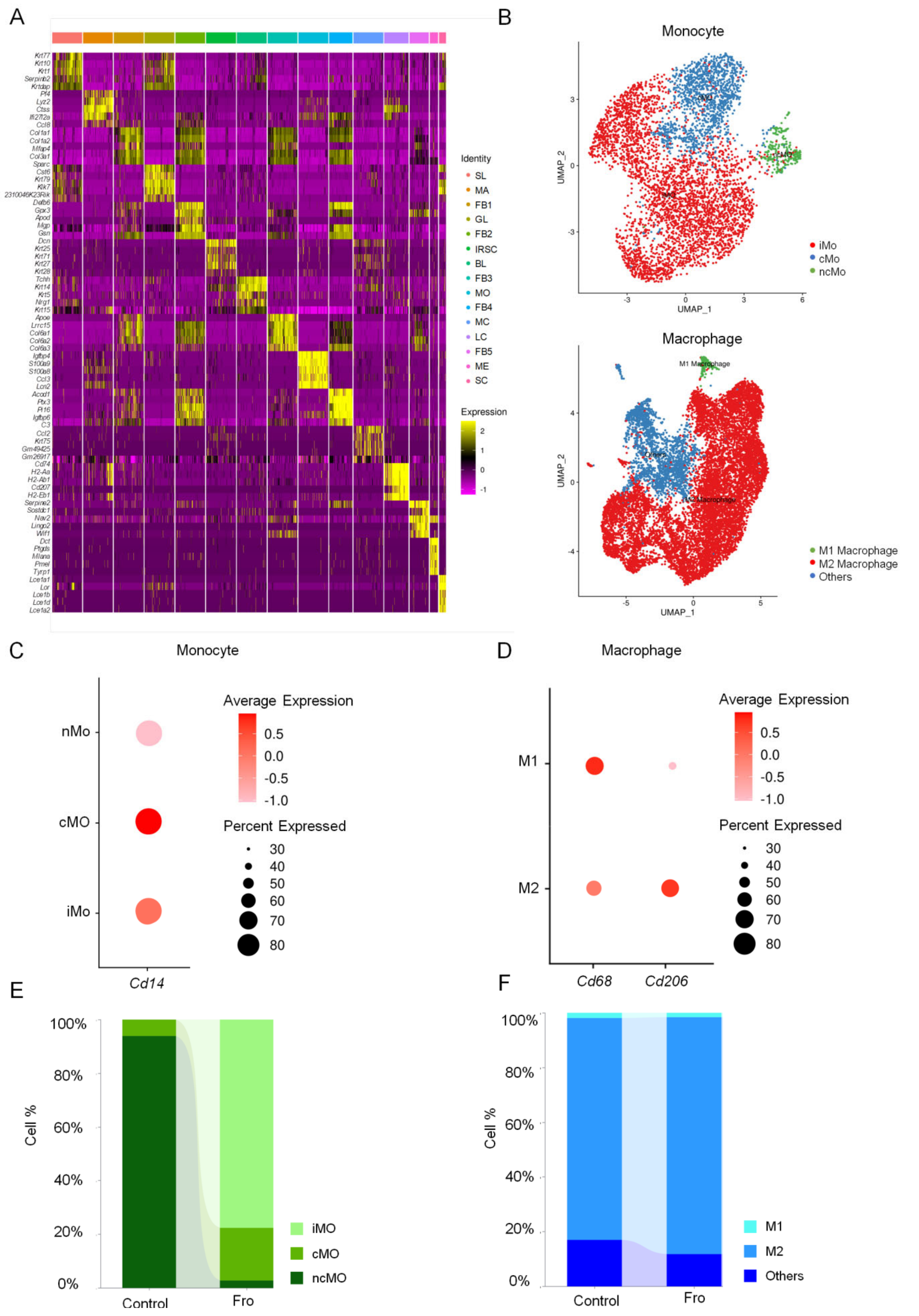

Figure S1. scRNA transcriptome profiles of skin samples from control and frostbite

model mice. (A) Heatmap of the normalized expression of the top 5 differentially expressed genes (DEGs) in each cell cluster according to the scRNA transcriptome profiles of skin samples from normal and frostbite model mice. Each row represents one DEG, and each column represents a single cell. SL: spinous layer cell; GL: granular layer cell; IRSC: inner root sheath cell; BL: basal layer cell; MC: medulla cell; ME: melanocyte; SC: stratum corneum cell; MA: macrophage; MO: monocyte; LC: Langerhans cell; FB1: fibroblast 1; FB2: fibroblast 2; FB3: fibroblast 3; FB4: fibroblast 4; FB5: fibroblast 5. (B) Subclustering of monocyte and macrophage in the skin samples of control and frostbite model mice. UMAP plots showing three subpopulations of monocyte including classical monocyte (cMo), intermediate monocyte (iMo), and non-classical monocyte (nMo), and two subpopulations of macrophage including M1 macrophage and M2 macrophage. Dot plot showing the expression of representative marker genes for different subtypes of monocyte (C) and macrophage (D). The statistical significance of cell type marker genes was determined using the Wilcoxon test with a BH-adjusted *p value* of <0.01. Histograms showing the proportions of different subtypes of monocyte (E) and macrophage (F) in the skin samples of control and frostbite model mice.

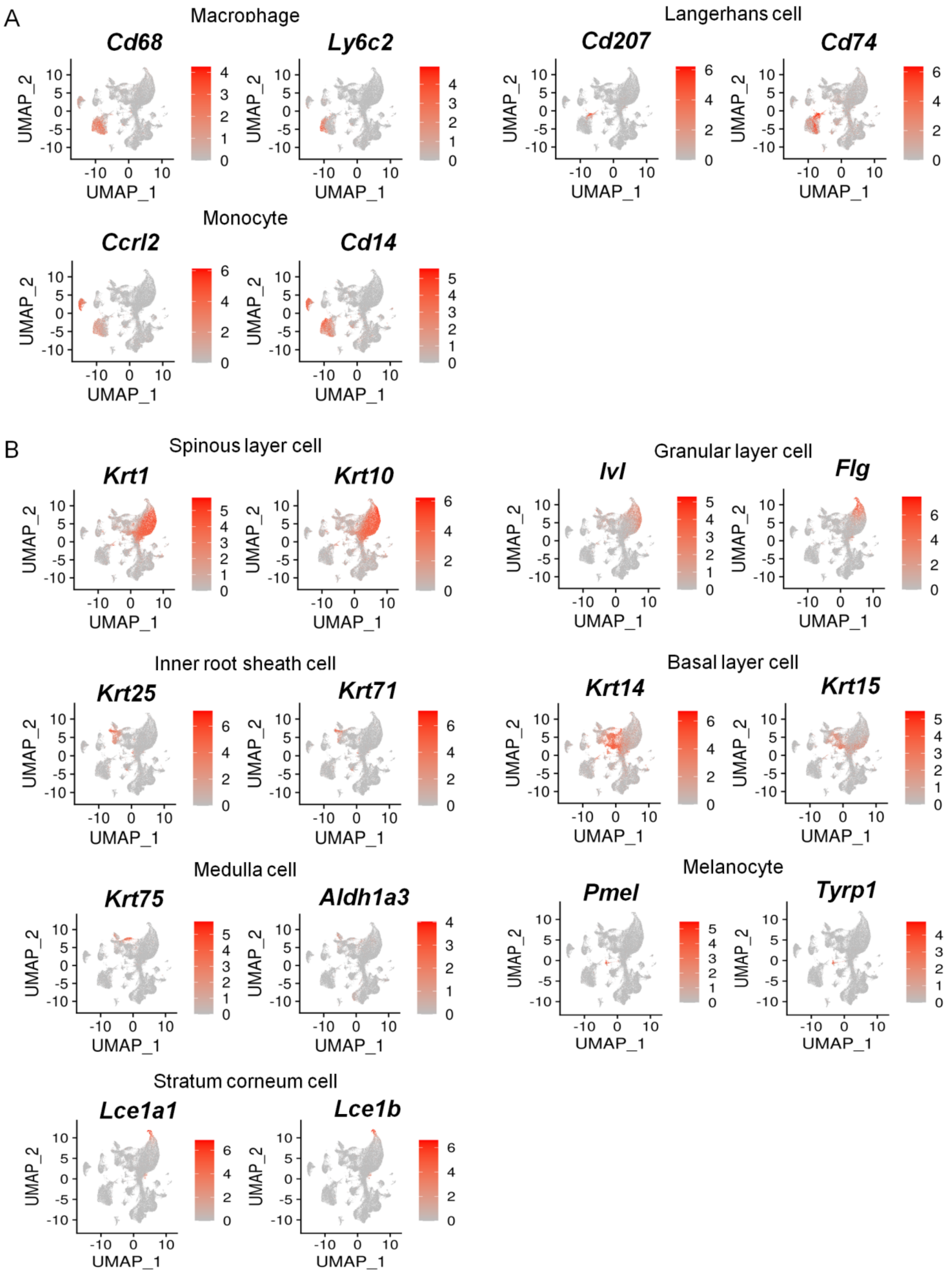

Figure S2. Distribution of representative marker genes for immune cells (A) and epidermal cells (B). The immune cell subtypes included Langerhans cells, monocytes, and macrophages; the epidermal cell subtypes included basal layer cells, spinous layer cells, granular layer cells, stratum corneum cells, melanocytes, inner root sheath cells, and medulla cells. The color scale indicates low expression (gray) to high expression (red).

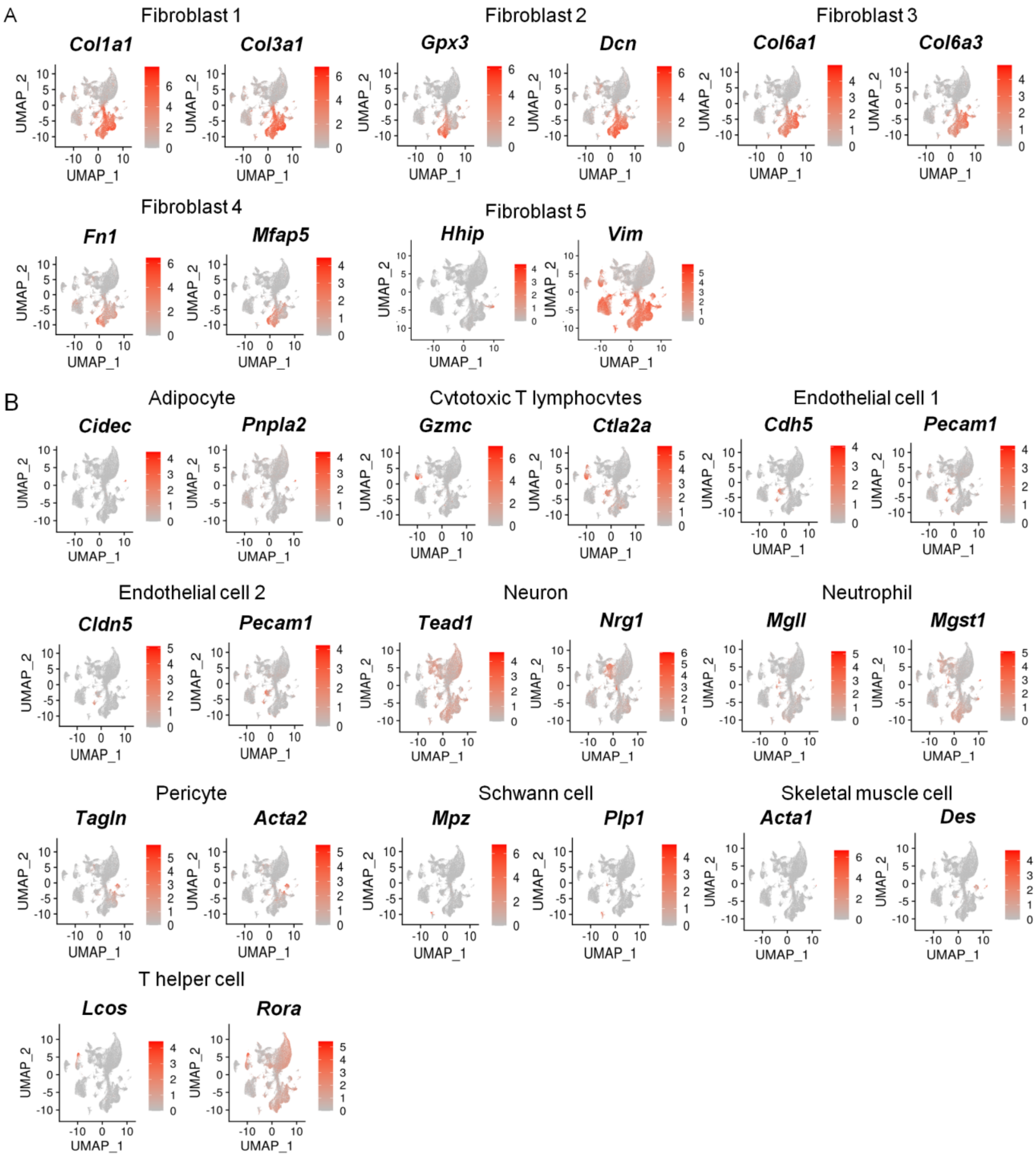

Figure S3. Distribution of representative marker genes for fibroblasts (A) and other cell types (B). The fibroblasts included five subtypes: fibroblast 1, fibroblast 2, fibroblast 3, fibroblast 4, and fibroblast 5. The color scale indicates low expression (gray) to high expression (red).

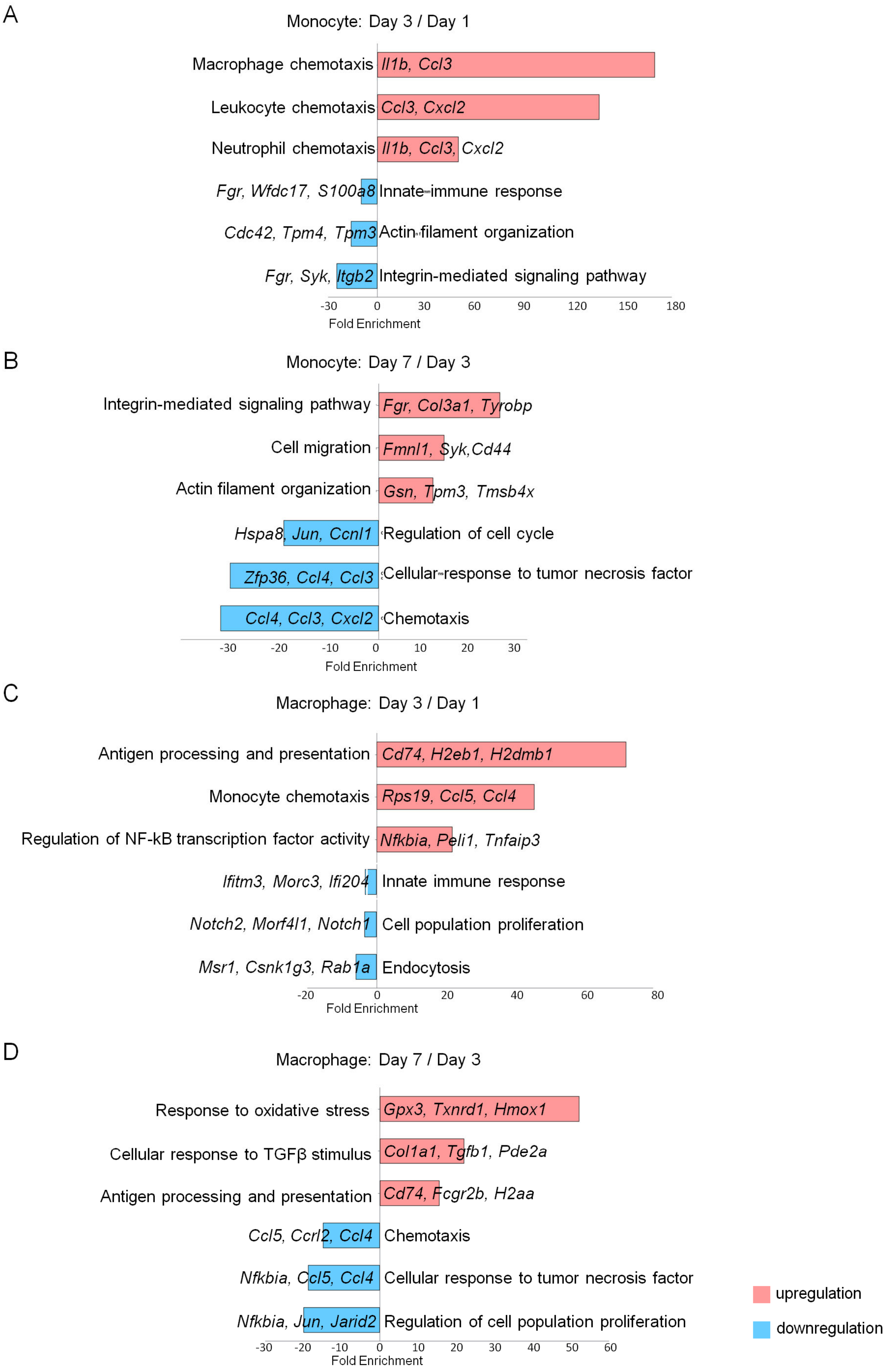

Figure S4. Functional analysis of differentially expressed genes (DEGs) in monocytes,

and macrophages between the control and frostbite groups at different days. Functional analysis of upregulated and downregulated DEGs in monocytes at day 3 compared to day 1 after frostbite (A) and at day 7 compared to day 3 after frostbite (B). Functional analysis of upregulated and downregulated DEGs in macrophages at day 3 compared to day 1 after frostbite (C) and at day 7 compared to day 3 after frostbite (D). Genes with a Benjamini–Hochberg (BH)-adjusted p value < 0.05 and log2FC > 0.25 (upregulated) or < -0.25 (downregulated) were identified as DEGs.

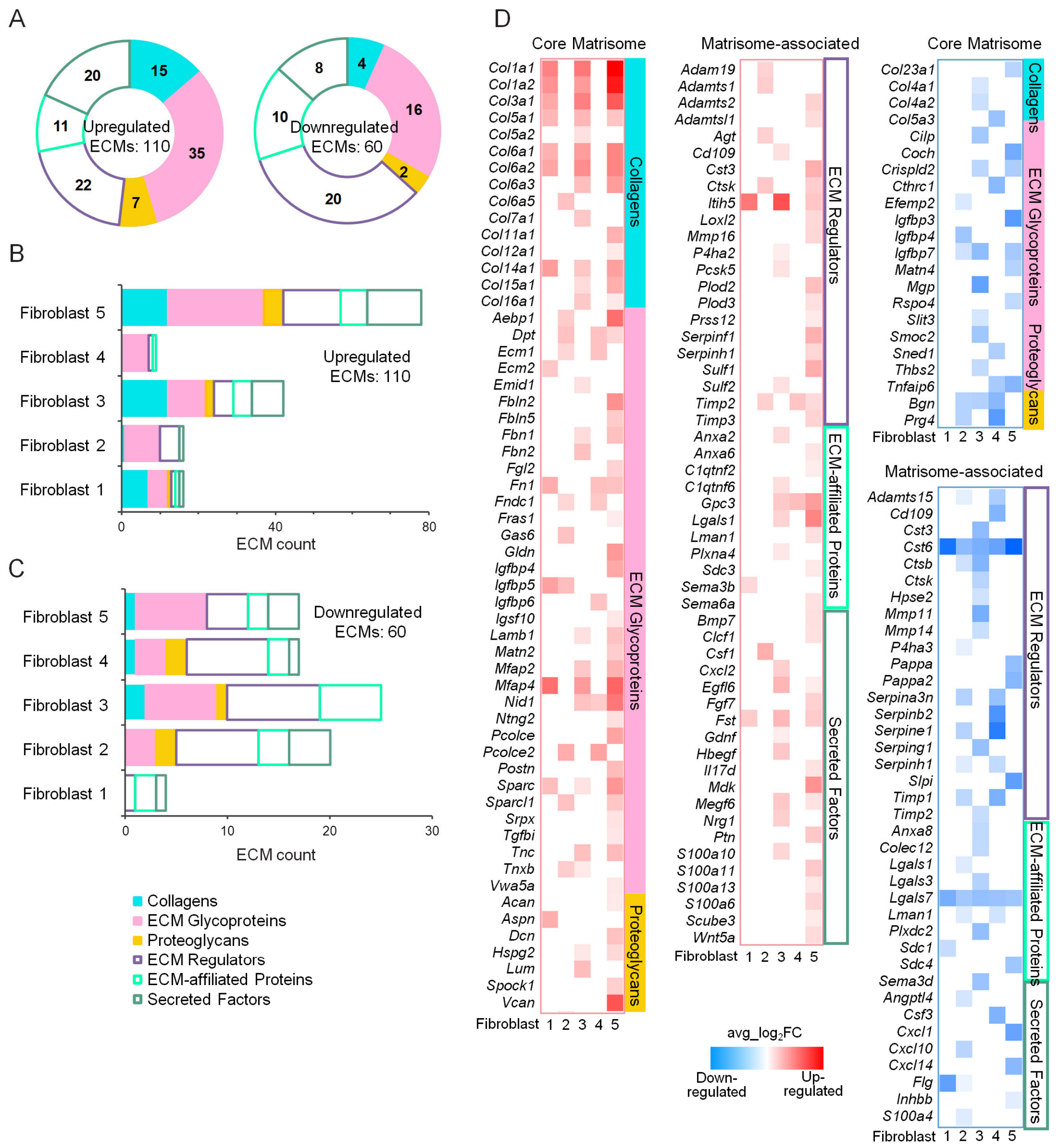

Figure S5. ECM characteristics in fibroblasts in the skin of frostbite model skin. (A) Pie charts showing the expression of six upregulated and downregulated ECM genes in fibroblasts between the day 7 frostbite and day 3 frostbite groups. Histograms show the detailed compositions of upregulated (B) and downregulated (C) ECM genes in five subtypes of fibroblasts between the day 7 frostbite and day 3 frostbite groups. Cyan, pink, and yellow represent collagens, ECM glycoproteins, and PGs, respectively; blue, green, and dark green represent ECM regulators, ECM-affiliated proteins, and secreted factors, respectively. Cyan,

pink, and yellow represent collagens, ECM glycoproteins, and glycoproteins, respectively; blue, green, and dark green represent ECM regulators, ECM-affiliated proteins, and secreted factors, respectively. (D) The red and blue bars indicate the log2 values of the upregulated and downregulated ECM genes in fibroblasts between the day 7 frostbite and day 3 frostbitegroups, respectively.

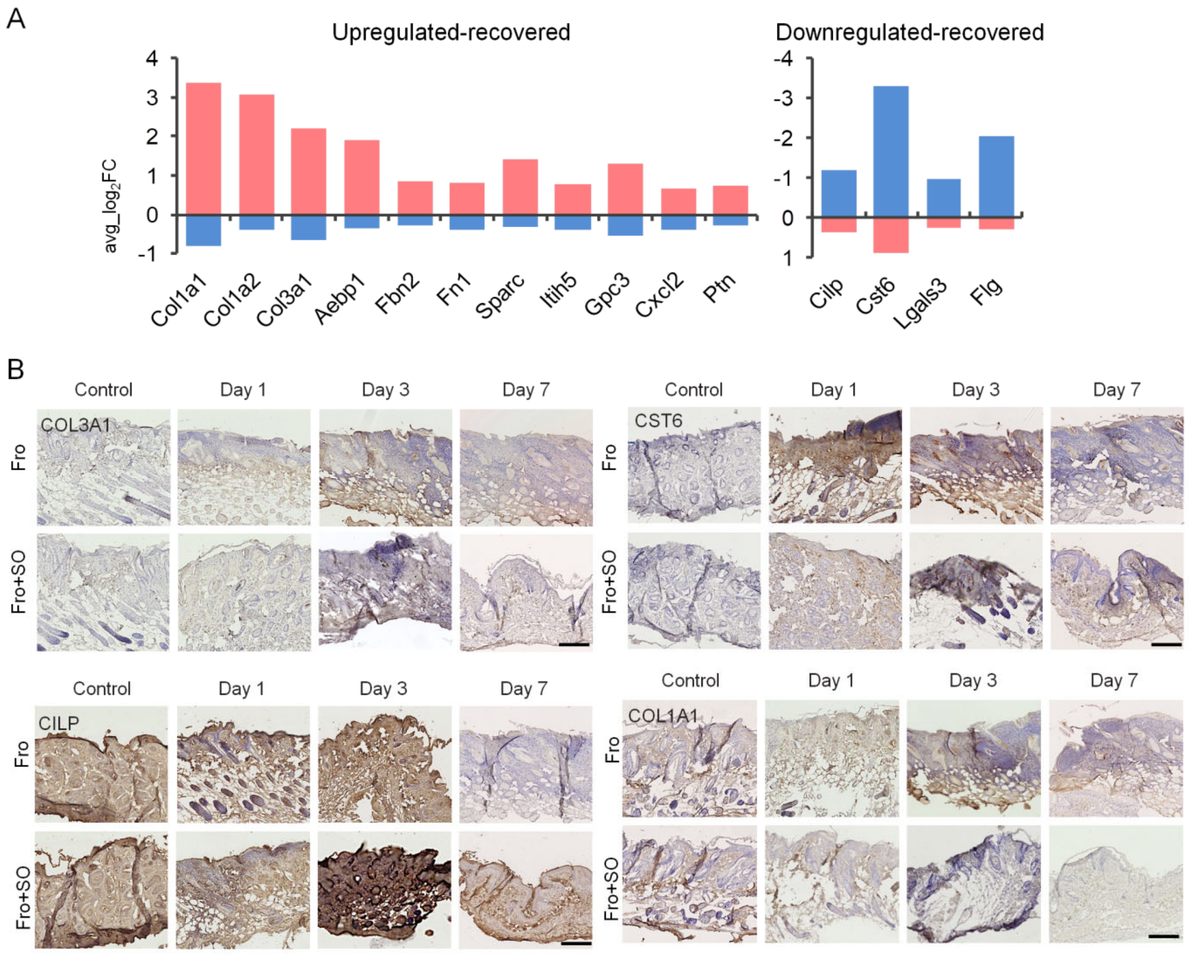

Figure S6. Recovery of the expression of ECM-associated components following skin organoids treatment. (A) Histograms showing the relative expression levels of ECM genes whose expression were upregulated or downregulated in mouse skin on day 7 after frostbite and recovered to normal levels by skin organoid treatment. (B) Immunohistochemical staining of COL1A1, COL3A1, CILP, and CST6 in mouse skin treated with and without skin organoids at 7 day after frostbite injury (scale bar: 500 µm). The experiment was repeated three times. ECM: extracellular matrix; Fro: frostbite; Fro+SO: frostbite with skin organoid treatment.
